# Characterization of the prevalence of excess weight in Brazil

**DOI:** 10.1186/s12889-022-13462-9

**Published:** 2022-06-06

**Authors:** Marcia Domênica Cunico Barancelli, Marcio Gazolla, Sergio Schneider

**Affiliations:** 1 Regional Development; Graduate Program in Regional Development (PPGDR) Federal Technological University of Paraná - UTFPR/Brazil, Via do Conhecimento - Km 1 (PR 493), Frarom neighbourhood, PO Box: 571, Pato Branco/PR, CEP 85503-390 Brazil; 2 Regional Development (2013) - PPGDR/UTFPR/Brazil, Pato Branco, Brazil; 3 Nursing Department - Federal Institute of Paraná - IFPR/Brazil, Palmas, Brazil; 4 Rural Development; Federal Technological University of Paraná - UTFPR/Brazil, Via do Conhecimento - Km 1 (PR 493), Frarom neighbourhood, PO Box: 571, Pato Branco/PR, CEP 85503-390 Brazil; 5 Academic Department of Agricultural Sciences (DAGRO) and Graduate Program in Regional Development (PPGDR), Pato Branco, Brazil; 6 Sociology of Rural Development and Food Studies at the Federal University of Rio Grande Do Sul (UFRGS), Av. João Pessoa, 31 – Centro, CEP: 90.040-000, Porto Alegre, Rio Grande Do Sul Brazil

**Keywords:** Food, Excess weight, Food and Nutrition Surveillance

## Abstract

**Introduction/Background:**

This work aims to analyse the prevalence of excess weight in Brazil to demonstrate the nutritional transition that is occurring. The data mobilized in the research are from the Food and Nutritional Surveillance System (FNSS).

**Materials and methods:**

This study employed a quantitative approach from the FNSS online secondary data survey through reports of nutritional status in different phases of life (child, adolescent, adult, elderly and pregnant), in different macroregions of the country (South, Southeast, Midwest, Northeast and North) and with a 12-year historical series (2008 to 2019).

**Results:**

In the adult life stage, there was a time trend of increasing excess weight in all regions of this historical series. The southern region of Brazil and the adult life stage had the highest national percentage of excess weight (69,1%) in 2019 and had the lowest percentage of eutrophy (29.3%) in the region in 2019. In the elderly life phase, in the South, Southeast and Midwest regions, excess weight was higher than the other outcomes in the time series, with the highest annual prevalence in the South region (58,6%) in 2019. In the adolescent life stage, there was a time trend of increasing excess weight in all regions, and excess weight had the highest prevalence in 2019 in the South (35,8%). The lowest prevalence rates of excess weight were in the following age groups: children aged 0 to <  2 years old and children aged 2 to <  5 years old. Additionally, it was in Group 5 to < 10 years old that the most critical prevalence of excess weight (35,07%) was found in 2018. However, malnutrition (low weight) persists, especially in the elderly and children. In the pregnancy life stage, there was a temporal trend of increase in excess weight in all regions, with higher percentages in 2019 in the South (53.5%) and Southeast (50.8%).

**Conclusion:**

Excess weight has shown increasing time trends in the adolescent, adult, elderly and pregnant life stages in all regions of Brazil, suggesting that public FNS policymakers should be more assertive in the planning and management of programs and actions to reduce the percentages of diseases.

**Supplementary Information:**

The online version contains supplementary material available at 10.1186/s12889-022-13462-9.

## Introduction

Food and eating practices are undergoing a rapid transformation process on a global scale that is largely dependent on how the food system and consumption habits of contemporary society are organized. On the one hand, the production of food, fibres and raw materials is homogenizing [1] and concentrating on a few grains (wheat, soy and rice) and proteins (poultry, pork and beef). On the other hand, consumption has shown growing trends in diets rich in high-calorie and fatty foods, which are often industrialized.

This picture is complemented by the growing concentration in the food distribution sector, as access to food is increasingly through supermarkets [2]. This system is organized in long chains that start in the input companies downstream of the farms and continue upstream until arriving on the supermarket shelves. Food production and distribution are concentrated in the hands of a few powerful global companies that vertically integrate the food chains [3]. As a result, the vertiginous growth of food-borne diseases, including obesity and overweight, can be perceived [4].

Manifestations of food insecurity (FI) denote a rapid nutritional transition [5] that corresponds to changes in nutritional patterns, modifications of people's diets and correlations with social, economic, demographic and health-related changes [6]. Although different aspects of nutrition and the economy of a country or region determine differences in the transition process, the common characteristics are the growth of ultra-processed diets [2] and reductions in complex carbohydrates, fibre and nutrients.

Populations go from malnutrition to obesity rapidly due to the complex relationships among three public health problems worldwide (obesity, malnutrition and climate change), which make up the "global syndemic” [7]. These three pandemics are characterized by poor nutrition, an agro-industrial model of production and inadequate eating habits of consumers as common determinants [7, 8]. In the Brazilian case, recent data released by the Brazilian Institute of Geography and Statistics (IBGE) through the Family Budget Survey (FBS) [9] indicate that the FI has increased again in recent years. Additionally, in this country, the increase was determined more due to public policies than by the factors indicated [10].

According to several analysts, overweight and obesity generate significant financial impacts on health systems [11] in both developing and developed countries, causing high levels of mortality from chronic noncommunicable diseases (CNCDs) [12, 13]. Obesity is not only one of these diseases but also a risk factor for others, such as heart disease, hypertension, diabetes, hypercholesterolemia, hyperlipidaemia and some forms of cancer. It is worth mentioning that obesity is one of the pre-existing conditions associated with mortality due to influenza H1N1 and currently COVID-19, given its impact on lung function [14, 15].

Obesity is defined as "an abnormal or excessive accumulation of body fat that can reach levels capable of affecting the health of the individual". The occurrence of these conditions is multifactorial, with poor nutrition, excessive consumption of ultra-processed foods, a sedentary lifestyle and presence of an endocrine disorder as the main factors. Projections indicate that in 2025, approximately 2.3 billion adults will be overweight, and more than 700 million people will be obese [16, 17]. In Brazil, studies [18, 19] have also highlighted this prevalence.

The objective of the work is to analyse the prevalence of excess weight in Brazil to demonstrate the nutritional transition that is occurring.

## Methods

### Data

This is an analytical epidemiological study with a quantitative approach as well as an ecological time-series study. It is based on a survey of secondary data from the FNSS website through reports of nutritional status at different stages of life (child, adolescent, adult, elderly and pregnant) and by macroregion of the country (Midwest, Northeast, North, Southeast and South) from a historical series of 12 years (2008 to 2019) conducted between July 2019 and July 2020.

The information presented in the FNSS comes from the daily routine of care provided by health professionals that is collected and consolidated with the assistance of the Basic Health Units (BHUs). The FNSS comprises data from the *DATA-SUS, Bolsa Família* and *E-SUS* platforms, defined as a Health Information System (HIS). This system enables the storage of data and the continuous generation of information on the nutritional status and food consumption of primary care users of the Unified Health System (UHS) [20].

Despite the efforts and investments to record FNSS data, the information remains underused by the management of action in food and nutrition, demonstrating in the Brazilian case study [21] that (1) there is an important number of people registered and accompanied [22], (2) there has been a significant increase [23] over the years in the number of people in the FNSS, (3) this fact reinforces the relevance of studies that analyse, synthesize and demonstrate the nutritional status at the level of the UHS using secondary data in primary care [24] through the system.

The FNSS consists of a unique national database that presents information on nutritional status and food consumption at all stages of life These data are collected by health professionals [25], who need constant training and an adequate institutional structure to strengthen this process under construction [26]. It is a set of administrative data from the entire country, with measures monitored over time [21], with monthly and weekly updates covering all regions, states and municipalities [25].

As a limitation in this study, the database, which is relevant at the level of follow-up in primary care of the SUS and extremely useful in identifying, for example, the serious nutritional situation of indigenous children [27], also has limited coverage, operation and infrastructure [23, 28, 29].

Nevertheless, the FNSS is useful and relevant, especially because it is complemented with other data sources, for example, from the DATA-SUS, Bolsa Família and E-SUS platforms, defined as the Health Information System and consolidated according to the guidelines of the Ministry of Health [25, 30], with protocols referenced in the study [31]. Details of the integration of the FNSS with other systems are published in another study as well [30].

In the data collection, it was possible to include the coverage of race/skin colour (white, black, yellow, brown and indigenous), peoples and communities (related to 20 communities: quilombola peoples, agroextractivists, caatingueiros, caiçaras, communities of background and closing of pasture, cerrado communities, extractivists, faxinalenses, geraizeiros, shellfish gatherers, pantaneiros, artisanal fishermen, Pomeranians, gypsy peoples, terreiro peoples, babassu coconut breakers, retreateiros, riverside dwellers, rubber tappers, evanteiros and others), in addition to schooling. Additionally, all levels of work were computed.

However, although the study involves a significant sample of SUS users (the public served in public health services), it does not include users of private health plans and the private health service, in addition to more vulnerable groups/peoples and communities that do not have access to primary health care.

Even so, it is noteworthy that the Brazilian Institute of Geography and Statistics (IBGE) showed that 71.5% of Brazilians, more than 150 million people, depended on the SUS in 2019 before the COVID-19 pandemic (9). This factor demonstrates the need for the researcher to examine this database as well as analyse and disseminate the relevant information to sensitize health managers as well as the population monitored. Doing so can serve as an instrument for planning actions to promote changes related to the promotion of health and food and nutrition security.

This work used the secondary database in the public domain of the FNSS website [32]. The data source was the FNSS website (http://sisaps.saude.gov.br/sisvan/relatoriopublico/index).

### Measures

For the evaluation of nutritional status, FNSS reports on BMI were used, with the unit of measurement being kg/m^2^. However, for each phase of life, the variables are presented according to the FNSS classification and follow the recommendations of the Ministry of Health (MH), which adopted the criteria of the World Health Organization (WHO). The data are parameterized by age in the health services, following the FNSS Technical Standard [33].

According to the FNSS [33], monitoring by phase of life is stratified as follows: children from 0 to < 2 years old and from 2 to < 5 (BMI x age) present the nutritional conditions of marked thinness, thinness, eutrophy, risk of overweight, overweight and obesity. In the life phase of 5 to < 10 years old, children have marked thinness, thinness, eutrophy, overweight, obesity and severe obesity. In adolescence (10 to < 20 years old), the conditions are marked thinness, thinness, eutrophy, overweight, obesity, and severe obesity. In the adult life phase (20 to < 60 years old), there is low weight, eutrophy, overweight, obesity grade I, obesity grade II and obesity grade III. In the elderly phase (older than 60 years old), there is low weight, eutrophy, and overweight. Finally, in the pregnancy life phase, there is low weight, eutrophy, overweight and obesity.

The nutritional condition risks of overweight, overweight, obesity, severe obesity, obesity grade I, obesity grade II and obesity grade III were grouped, and they are represented in this study as “excess weight”.

People with a body mass index (BMI) equal to or greater than 30 kg/m2 are considered obese, and those with a BMI between 25 and 30 kg/m2 are considered overweight [34].

Supplementary Table 1 designates indicators of different nutritional conditions and life stages.

Supplementary Table 2 shows the total number of people monitored by SISVAN in the historical series (n total of the study).

### Statistical approach

Data were extracted from the FNSS database through public reports available on the Internet and stored using Microsoft Office Excel ® 2016. They were arranged descriptively in absolute (n) supplementary tables and relative (%) frequencies using tables. The 228 reports generated were compiled and analysed for database formatting for prevalence analysis and trends in the time series.

In the statistical analysis, a linear regression model was built for each time series, and a two-sided t test was performed for the model parameters with a 95% confidence level.

In a linear regression model, the angular coefficient represents the slope of the regression line. When this coefficient is positive, it means that the line is increasing (proportions tend to increase in the historical series). When it is negative, it means that the line is decreasing (proportions decrease over the years).

The *p *values indicate the results of the two-sided t test, which checks whether the angular coefficients are significantly different from zero. At the 5% significance level, when *p* < 0.025, the angular coefficient is said to be significantly different from 0, and the regression line has an increasing or decreasing trend. When *p* > 0.025, the angular coefficient is said to be statistically equal to 0, and the regression line tends to remain horizontal (no increase or a decreasing trend).

The statistical analyses were performed using R software, and the codes used in the tables and graphs (supplementary material) can be found at the following address for access and possible reproduction of the work: https://github.com/Marcia-Domenica/Nutritional-status-of-Brazilians/blob/Artigo/Analises1.R

## Disclosure of ethical standards

This study is part of a thesis project entitled "The relationship between obesity and food and nutritional security based on the interpretation of FNSS data: What a public policy can disclose." The present study was conducted in accordance with the guidelines established in the Declaration of Helsinki (research that uses information in the public domain, Ministry of Health, National Health Council, resolution no. 510, of April 7, 2016 [32].

## Results

### Child life phase 0 to < 2 years old

Table 1 demonstrates the prevalence and annual time trend of the nutritional status of children aged 0 to < 2 years old by Brazilian region from 2008 to 2019. There is a 95% confidence level.

**Table 1:** Prevalence and annual time trend of nutritional status of children aged 0 to < 2 years by Brazilian region, from 2008 to 2019. There is a 95% confidence level

Regions	Nutritional status	Ano												*Annual average variation	**P value	Trend
		**2008**	**2009**	**2010**	**2011**	**2012**	**2013**	**2014**	**2015**	**2016**	**2017**	**2018**	**2019**			
		**%**	**%**	**%**	**%**	**%**	**%**	**%**	**%**	**%**	**%**	**%**	**%**			
North	Marked thinness	5.6	4.9	5.1	4.4	4.2	4.5	4.3	3.6	3.8	3.6	2.9	4.3	-0.165	0.001	Decrease
	Thinness	3.1	3.1	3.1	2.9	3.0	2.9	2.8	2.9	2.9	3.2	2.6	3.7	0.010	0.674	
	Eutrophy	47.5	48.3	48.7	49.1	49.7	47.1	45.0	49.2	51.7	50.2	50.1	53.1	0.338	0.049	
	Excess of weight	43.9	43.7	43.1	43.6	43.1	45.5	47.9	44.3	41.6	43.1	44.4	38.9	-0.183	0.328	
Northeast	Marked thinness	6.6	6.4	6.0	5.8	5.0	5.1	5.0	3.8	4.3	3.7	3.2	4.4	-0.281	< 0,001	Decrease
	Thinness	3.2	3.1	3.1	3.0	2.9	3.0	3.0	2.7	2.9	2.7	2.8	3.5	-0.015	0.452	
	Eutrophy	42.9	43.3	44.1	43.9	45.9	44.8	43.4	47.9	49.2	47.9	49.0	50.9	0.689	0.000	Increase
	Excess of weight	47.3	47.1	46.8	47.2	46.2	47.2	48.6	45.5	43.7	45.7	45.0	41.1	-0.393	0.009	Decrease
Midwest	Marked thinness	5.8	5.2	5.0	4.7	4.4	4.5	4.8	3.6	3.6	3.3	2.9	4.4	-0.192	0.001	Decrease
	Thinness	3.4	3.6	3.5	3.4	3.0	2.9	3.0	3.0	3.0	2.9	2.8	4.1	-0.017	0.633	
	Eutrophy	52.9	55.1	53.5	56.1	56.7	54.3	52.8	55.9	58.1	56.6	56.1	58.6	0.348	0.018	Increase
	Excess of weight	38.0	36.2	38.0	35.8	36.0	38.4	39.4	37.5	35.4	37.2	38.1	32.9	-0.139	0.367	
Southeast	Marked thinness	4.5	4.1	4.1	3.5	3.5	3.5	3.7	2.8	2.9	2.8	2.6	3.3	-0.147	< 0,001	Decrease
	Thinness	2.7	2.7	2.7	2.6	2.7	2.7	2.8	2.7	2.7	2.6	2.5	3.3	0.015	0.375	
	Eutrophy	54.1	56.1	54.9	56.2	57.5	54.0	54.3	57.6	59.1	58.4	58.3	60.1	0.435	0.004	Increase
	Excess of weight	38.6	37.1	38.3	37.7	36.2	39.8	39.2	36.8	35.4	36.2	36.7	33.3	-0.303	0.032	
South	Marked thinness	3.2	2.7	2.6	2.4	2.2	2.2	2.2	1.7	1.8	1.7	1.5	1.9	-0.127	< 0,001	Decrease
	Thinness	2.4	2.4	2.2	2.2	2.3	2.2	2.2	2.1	2.1	2.1	2.0	2.4	-0.019	0.111	
	Eutrophy	57.9	59.1	57.8	59.3	60.9	57.2	57.7	59.6	61.6	60.0	59.6	60.4	0.195	0.089	
	Excess of weight	36.6	35.7	37.4	36.1	34.6	38.4	37.9	36.5	34.5	36.3	36.9	35.3	-0.050	0.642	

*Note*: * Annual average variation is calculated by the linear regression method; **P value indicates the results of the two-sided t test

Extreme thinness showed an annual time trend of a decline in all regions; the South had the lowest prevalence (1,5%) in 2018, and the Northeast had the highest prevalence (6,6%) in 2008.

The nutritional condition thinness, despite having low prevalence percentage values, did not show a decreasing trend in any region; the South had the lowest prevalence (2,0%) in 2018, and the Midwest had the highest prevalence (4,1%) in 2019.

The eutrophic nutritional condition showed a temporal trend to increase in the Northeast, Midwest and Southeast regions. However, although the Northeast region had a temporal tendency to increase, it had the lowest prevalence of eutrophy, with a lower percentage in 2008 (42.9%). Additionally, the highest prevalence of eutrophy was in the South at 61.6% in 2016.

Table 1 Prevalence and annual time trend of nutritional status of children aged 0 to < 2 years old by Brazilian region from 2008 to 2019. There is a 95% confidence level.

The central-western, southeastern, and southern regions had higher percentages of eutrophy than the northern and northeastern regions.

The highest prevalence of excess weight was in the Northeast region (48.6%) in 2014. Also, from 2008 to 2014, excess weight had higher percentages than the eutrophic outcome in this region. However, the Northeast is the only region that showed a decreasing trend in the time series nutritional condition excess weight. The North had the second highest prevalence of excess weight (47.9) in 2014; however, this region showed no variation in the temporal trend.

The lowest prevalence of excess weight was in the Midwest (32.9%) and Southeast (33.3%) in 2019.

### Child life phase 2 to < 5 years old

According to Table 2, the prevalence and annual temporal trend of the nutritional status of children aged 2 to < 5 years old showed the lowest prevalence and a decreasing trend in the South region in relation to thinness (1,8%) and marked thinness (1,4%) in 2018; however, in the nutritional condition excess weight, this region had the highest prevalence (37,2%) in 2015.

**Table 2:** Prevalence and annual time trend of nutritional status of children aged 2 to < 5 years by Brazilian region, from 2008 to 2019. There is a 95% confidence level

Regions	Nutritional status	Ano												*Annual average variation	**P value	Trend
		**2008**	**2009**	**2010**	**2011**	**2012**	**2013**	**2014**	**2015**	**2016**	**2017**	**2018**	**2019**			
		**%**	**%**	**%**	**%**	**%**	**%**	**%**	**%**	**%**	**%**	**%**	**%**			
North	Marked thinness	4.4	4.2	4.2	4.0	3.7	3.9	3.5	3.1	3.4	2.9	2.6	3.3	-0.142	< 0,001	Decrease
	Thinness	3.8	3.7	3.9	3.8	3.8	3.8	3.5	3.6	3.5	3.7	3.1	4.1	-0.015	0.475	
	Eutrophy	63.3	63.0	63.5	62.8	65.0	63.3	63.5	65.4	64.3	66.3	66.8	67.8	0.391	< 0,001	Increase
	Excess of weight	28.4	29.2	28.5	29.3	27.5	29.0	29.5	27.9	28.8	27.0	27.5	24.7	-0.233	0.028	
Northeast	Marked thinness	5.3	5.1	5.2	5.0	4.7	4.5	4.2	3.7	4.2	3.7	3.2	3.8	-0.180	< 0,001	Decrease
	Thinness	4.1	4.1	4.3	4.1	4.1	4.0	3.8	3.8	3.9	3.8	3.6	4.2	-0.033	0.043	
	Eutrophy	58.9	58.6	58.5	58.1	59.3	59.0	57.7	59.4	58.2	59.9	60.4	61.6	0.194	0.024	Increase
	Excess of weight	31.7	32.3	32.0	32.8	32.0	32.5	34.2	33.1	33.7	32.7	32.8	30.5	0.019	0.822	
Midwest	Marked thinness	4.6	4.4	4.4	4.5	4.3	4.1	4.3	3.1	3.4	2.8	2.3	2.9	-0.199	< 0,001	Decrease
	Thinness	3.8	3.6	3.7	3.5	3.3	3.2	3.2	3.1	3.1	3.1	2.8	3.6	-0.049	0.030	
	Eutrophy	61.8	61.6	61.6	61.4	61.9	62.0	60.8	62.4	62.3	64.6	64.7	66.7	0.382	0.003	Increase
	Excess of weight	29.9	30.5	30.4	30.7	30.5	30.7	31.7	31.4	31.3	29.5	30.1	26.7	-0.134	0.228	
Southeast	Marked thinness	3.4	3.3	3.5	3.2	3.2	3.3	3.3	2.8	2.9	2.6	2.8	2.9	-0.066	0.001	Decrease
	Thinness	2.9	2.9	3.0	2.8	2.8	2.8	2.9	2.8	2.8	2.8	2.6	3.3	0.005	0.737	
	Eutrophy	62.5	62.5	62.1	61.1	61.5	60.1	60.3	61.1	61.3	62.4	62.4	64.3	0.079	0.435	
	Excess of weight	31.3	31.4	31.5	33.0	32.5	33.9	33.6	33.4	33.0	32.2	32.2	29.5	-0.018	0.872	
South	Marked thinness	2.6	2.4	2.4	2.2	2.2	2.3	2.3	1.8	2.0	1.6	1.4	1.5	-0.101	< 0,001	Decrease
	Thinness	2.2	2.1	2.2	2.1	2.1	2.0	2.1	1.9	2.0	2.0	1.8	2.1	-0.025	0.004	Decrease
	Eutrophy	61.0	60.8	60.8	60.5	60.7	58.8	58.7	59.2	60.2	61.2	61.4	63.6	0.106	0.368	
	Excess of weight	34.2	34.7	34.6	35.2	35.0	36.9	37.0	37.2	35.8	35.1	35.4	32.9	0.020	0.863	

*Note*: * Annual average variation is calculated by the linear regression method; **P value indicates the results of the two-sided t test

Table 2 Prevalence and annual time trend of nutritional status of children aged 2 to < 5 years old by Brazilian region from 2008 to 2019. There is a 95% confidence level.

Extreme thinness was the only nutritional condition that showed a decreasing temporal trend in all Brazilian regions, with the Northeast region having the highest prevalence (5.3%) in 2008 and the South having the lowest prevalence.

In the midwestern, northern and northeastern regions, there was a time trend towards increasing eutrophy. In the north, the highest prevalence was 67.8% in 2019, followed by the midwestern region, with a prevalence of 66.7%.

Despite the increase in the time series of the eutrophic outcome, the Northeast region had the lowest prevalence of this condition at the national level as well as the highest prevalence of thinness and accentuated thinness. Excess weight remained, with prevalence ranging from 30.5 to 34.2 in the historical series.

### Child life phase 5 to < 10 years old

Among the child life stages, the 5 to < 10 years old group had the most critical prevalence of excess weight. Table 3 shows that for eutrophia, the temporal trend decreased in the Northeast, Southeast and South regions. In the North region, eutrophy remained stable, with a higher prevalence of 72.5% in 2019. The lowest prevalence of this nutritional condition was in the South region in 2015 (62.2%) and 2019 (63.3%).

**Table 3:** Prevalence and annual time trend of nutritional status of children aged 5 to < 10 years by Brazilian region, from 2008 to 2019. There is a 95% confidence level

Regions	Nutritional status	Ano												*Annual average variation	**P value	Trend
		**2008**	**2009**	**2010**	**2011**	**2012**	**2013**	**2014**	**2015**	**2016**	**2017**	**2018**	**2019**			
		**%**	**%**	**%**	**%**	**%**	**%**	**%**	**%**	**%**	**%**	**%**	**%**			
North	Marked thinness	3.8	3.9	3.7	3.7	3.4	3.6	3.2	2.6	2.7	2.6	2.2	2.8	-0.148	< 0,001	Decrease
	Thinness	3.7	3.6	3.8	3.9	3.7	3.9	3.6	3.5	3.4	3.4	3.2	3.8	-0.030	0.110	
	Eutrophy	72.2	72.0	72.7	72.5	72.9	72.0	72.4	72.4	71.5	72.0	72.4	72.5	-0.017	0.604	
	Excess of weight	20.3	20.4	19.8	19.9	20.0	20.5	20.8	21.5	22.3	22.1	22.3	20.9	0.195	0.004	increase
Northeast	Marked thinness	4.2	4.2	4.3	4.1	3.7	3.6	3.3	2.8	3.0	2.8	2.3	2.7	-0.182	< 0,001	Decrease
	Thinness	4.1	4.2	4.4	4.1	4.1	4.0	3.7	3.7	3.8	3.7	3.5	4.0	-0.052	0.006	Decrease
	Eutrophy	67.9	67.2	66.3	65.4	65.7	65.5	64.9	65.0	64.0	64.8	64.8	65.3	-0.248	0.001	Decrease
	Excess of weight	23.8	24.4	25.1	26.4	26.6	26.8	28.1	28.5	29.2	28.7	29.3	28.0	0.483	< 0,001	Increase
Midwest	Marked thinness	3.3	3.3	3.5	3.2	3.2	3.2	3.2	2.2	2.3	2.1	1.7	2.0	-0.158	< 0,001	Decrease
	Thinness	3.7	3.3	3.3	3.2	3.1	3.0	3.0	2.9	2.9	2.9	2.7	3.3	-0.055	0.009	Decrease
	Eutrophy	68.3	68.1	67.7	67.7	67.5	67.3	65.9	66.2	66.1	67.4	67.1	67.5	-0.117	0.065	
	Excess of weight	24.7	25.3	25.5	25.9	26.2	26.6	27.8	28.7	28.7	27.7	28.5	27.2	0.332	< 0,001	Increase
Southeast	Marked thinness	2.6	2.7	2.8	2.5	2.3	2.4	2.3	1.9	1.9	1.9	1.8	1.9	-0.092	< 0,001	Decrease
	Thinness	2.7	2.8	2.8	2.6	2.6	2.6	2.6	2.5	2.6	2.6	2.5	3.0	-0.002	0.852	
	Eutrophy	69.8	68.9	68.4	67.0	66.7	65.9	65.7	64.5	64.4	64.5	64.5	65.2	-0.481	< 0,001	Decrease
	Excess of weight	24.9	25.6	26.1	27.9	28.3	29.1	29.4	31.0	31.1	31.0	31.2	29.9	0.576	< 0,001	Increase
South	Marked thinness	2.0	2.0	2.0	1.7	1.7	1.8	1.7	1.2	1.3	1.2	1.0	1.0	-0.099	< 0,001	Decrease
	Thinness	2.0	2.1	2.1	1.9	2.0	1.8	1.8	1.7	1.7	1.7	1.7	1.9	-0.032	0.002	Decrease
	Eutrophy	67.8	67.2	66.3	65.9	64.8	63.4	63.2	62.2	62.3	62.4	62.3	63.3	-0.514	< 0,001	Decrease
	Excess of weight	28.22	28.72	29.67	30.52	31.51	33.01	33.25	34.9	34.65	34.69	35.07	33.79	0.646	< 0,001	Increase

*Note*: * Annual average variation is calculated by the linear regression method; **P value indicates the results of the two-sided t test

Excess weight showed a temporal trend of increasing in all regions of the country. The highest prevalence (35.07%) of excess weight was found in the South region in 2018.

Table 3 Prevalence and annual time trend of nutritional status of children aged 5 to < 10 years old by Brazilian region from 2008 to 2019. There is a 95% confidence level.

There was a nutritional transition detected, as there was also a decrease in extreme thinness in all regions, with the nutritional condition eutrophic remaining stable only in the North region. Thus, in the Northeast, Midwest, Southeast, and South regions, an increase occurred in excess weight. There was a transition from malnutrition to obesity in four of the five macroregions of Brazil.

### Adolescent life phase (10 to < 20 years old)

In the adolescent life stage, according to Table 4, there was a drop in the time trend of the nutritional condition extreme thinness in the South (0.5%) and Southeast (0.9%) regions, with the lowest prevalence in 2018.

**Table 4:** Prevalence and annual time trend of nutritional status of adolescents (10 to < 20 years) by Brazilian region, 2008 to 2019. There is a 95% confidence level

Regions	Nutritional status	Ano												*Annual average variation	**P value	Trend
		**2008**	**2009**	**2010**	**2011**	**2012**	**2013**	**2014**	**2015**	**2016**	**2017**	**2018**	**2019**			
		**%**	**%**	**%**	**%**	**%**	**%**	**%**	**%**	**%**	**%**	**%**	**%**			
North	Marked thinness	2.1	1.1	1.3	1.0	1.3	1.3	1.5	1.1	1.2	1.1	0.9	1.1	-0.049	0.070	
	Thinness	3.1	2.5	2.3	2.4	2.8	2.9	2.7	2.8	2.9	2.8	2.6	3.0	0.022	0.283	
	Eutrophy	78.9	80.7	80.5	79.9	77.7	76.7	75.7	73.9	73.6	73.4	72.1	72.1	-0.862	< 0,001	Decrease
	Excess of weight	15.9	15.7	15.9	16.8	18.2	19.1	20.1	22.2	22.4	22.7	24.4	23.8	0.889	< 0,001	Increase
Northeast	Marked thinness	2.6	1.1	1.7	1.0	1.3	1.5	1.5	1.3	1.3	1.2	1.1	1.4	-0.051	0.153	
	Thinness	3.6	2.8	2.7	2.6	3.1	3.4	3.2	3.4	3.4	3.4	3.3	3.7	0.053	0.058	
	Eutrophy	78.1	81.4	79.8	79.1	76.7	75.5	74.2	73.1	72.4	72.1	70.1	69.6	-1.031	< 0,001	Decrease
	Excess of weight	15.7	14.7	15.8	17.2	18.9	19.7	21.0	22.3	22.8	23.2	25.5	25.3	1.029	< 0,001	Increase
Midwest	Marked thinness	2.1	1.0	1.1	1.0	1.1	1.3	1.5	1.1	1.1	1.0	0.8	1.0	-0.050	0.075	
	Thinness	3.0	2.5	2.4	2.4	2.6	2.7	2.7	2.9	2.9	2.9	2.6	3.0	0.029	0.100	
	Eutrophy	74.9	76.7	76.8	74.9	72.1	71.3	70.4	68.6	67.8	67.9	66.5	65.8	-1.051	< 0,001	Decrease
	Excess of weight	20.0	19.8	19.7	21.7	24.1	24.7	25.5	27.4	28.2	28.2	30.0	30.2	1.073	< 0,001	Increase
Southeast	Marked thinness	1.9	1.3	1.2	1.0	1.0	1.2	1.2	1.0	1.0	1.1	0.9	1.0	-0.050	0.015	Decrease
	Thinness	3.0	2.5	2.4	2.3	2.5	2.6	2.6	2.7	2.7	2.8	2.7	2.9	0.021	0.217	
	Eutrophy	75.4	76.5	75.5	74.3	72.3	71.2	70.4	68.2	67.3	67.2	65.8	65.0	-1.109	< 0,001	Decrease
	Excess of weight	19.7	19.7	20.9	22.3	24.2	25.0	25.7	28.2	29.0	29.0	30.6	31.1	1.137	< 0,001	Increaseo
South	Marked thinness	1.4	1.0	1.0	0.8	0.8	1.0	1.1	0.7	0.7	0.6	0.5	0.6	-0.061	< 0,001	Decrease
	Thinness	2.1	1.6	1.5	1.5	1.7	1.7	1.7	1.8	1.8	1.9	1.8	2.0	0.022	0.170	
	Eutrophy	72.2	72.6	71.3	70.0	68.6	67.4	66.6	64.5	63.9	63.3	62.3	61.6	-1.082	< 0,001	Decrease
	Excess of weight	24.3	24.8	26.2	27.7	28.9	30.0	30.6	33.0	33.6	34.2	35.4	35.8	1.122	< 0,001	Increase

Note: * Annual average variation is calculated by the linear regression method; **P value indicates the results of the two-sided t test

Eutrophy showed a decreasing temporal trend in all regions. The South had the lowest prevalence (61.6%), whereas the North had the highest prevalence (72.1%) in 2019.

Table 4 Prevalence and annual time trend of nutritional status of adolescents (10 to < 20 years old) by Brazilian region from 2008 to 2019. There is a 95% confidence level.

There was a time trend of increasing excess weight in all regions. Excess weight had the highest prevalence in 2019 in the South (35,8%) region, followed by the Southeast (31,1%), and Midwest (30,2%) regions.

### Adult life phase

There was a decreasing temporal trend in underweight in all regions, with the lowest prevalence (1,6%) in the South region in 2019, according to Table 5. Eutrophy showed a decrease in the time trend in all regions, with the lowest prevalence in the South (29,3%), Midwest (32,4%) and Southeast (32,7%) regions in 2019. The highest prevalence of eutrophy occurred in the Northeast (54,4%) and North (53,1%) regions in 2008; however, there was also a decreasing temporal trend, with a prevalence of 36,9% (North and Northeast) in 2019.

**Table 5:** Prevalence and annual time trend of adult nutritional status by Brazilian region from 2008 to 2019. There is a 95% confidence level

Regions	Nutritional status	Ano												*Annual average variation	**P value	Trend
		**2008**	**2009**	**2010**	**2011**	**2012**	**2013**	**2014**	**2015**	**2016**	**2017**	**2018**	**2019**			
		**%**	**%**	**%**	**%**	**%**	**%**	**%**	**%**	**%**	**%**	**%**	**%**			
North	Low weight	5.5	4.3	4.6	4.0	3.5	3.7	3.7	2.8	2.9	2.5	2.4	2.5	-0.254	< 0,001	Decrease
	Eutrophy	53.1	52.7	52.1	50.6	47.4	46.1	44.2	40.7	41.0	40.1	37.9	36.9	-1.625	< 0,001	Decrease
	Excess of weight	41.4	43.0	43.3	45.4	49.1	50.2	52.1	56.5	56.1	57.3	59.7	60.6	1.879	< 0,001	Increase
Northeast	Low weight	6.4	4.8	5.2	4.2	3.8	3.8	3.5	2.9	3.1	2.8	2.7	2.9	-0.289	< 0,001	Decrease
	Eutrophy	54.4	54.2	52.5	50.8	47.6	45.7	43.3	41.2	40.9	39.7	37.9	36.9	-1.747	< 0,001	Decrease
	Excess of weight	39.2	41.0	42.4	45.1	48.6	50.5	53.2	55.9	56.0	57.5	59.5	60.2	2.037	< 0,001	Increase
Midwest	Low weight	5.4	4.0	3.9	3.5	3.3	3.4	3.4	3.0	2.7	2.6	2.4	2.4	-0.217	< 0,001	Decrease
	Eutrophy	49.1	48.2	48.3	45.8	42.2	41.0	39.5	36.2	35.7	34.7	33.7	32.4	-1.680	< 0,001	Decrease
	Excess of weight	45.5	47.8	47.8	50.7	54.6	55.7	57.2	60.8	61.6	62.7	64.0	65.2	1.897	< 0,001	Increase
Southeast	Low weight	5.1	4.2	3.9	3.5	3.2	3.3	3.2	2.7	2.7	2.6	2.4	2.4	-0.210	< 0,001	Decrease
	Eutrophy	47.3	46.4	44.8	43.0	40.2	38.7	37.5	35.4	34.9	34.8	33.7	32.7	-1.395	< 0,001	Decrease
	Excess of weight	47.7	49.5	51.3	53.6	56.6	58.0	59.3	61.9	62.3	62.6	63.9	64.8	1.605	< 0,001	Increase
South	Low weight	3.9	3.9	3.7	3.4	2.6	2.7	2.7	1.9	2.0	1.8	1.6	1.6	-0.235	< 0,001	Decrease
	Eutrophy	44.0	43.2	42.0	40.0	37.9	36.2	35.1	32.3	32.4	31.1	30.0	29.3	-1.444	< 0,001	Decrease
	Excess of weight	52.2	52.8	54.2	56.7	59.5	61.1	62.1	65.8	65.6	67.1	68.3	69.1	1.679	< 0,001	Increase

*Note*: * Annual average variation is calculated by the linear regression method; **P value indicates the results of the two-sided t test

The research showed that in the adult phase of life, there were the most critical findings in relation to the decrease in eutrophy and increases in excess weight in relation to the other phases of life because there are data showing increases in food insecurity situations in all regions.

Table 5 Prevalence and annual time trend of adult nutritional status by Brazilian region from 2008 to 2019. There is a 95% confidence level.

There was a time trend of increasing excess weight in all regions.

Excess weight had its highest prevalence in the South (69,1%) region, followed by the Midwest (65,2%), Southeast (64,8%), North (60,6%) and Northeast (60,2%) regions in 2019.

In the adult life stage, there was the lowest prevalence of eutrophy in comparison to excess weight compared with the other life stages. When considering the nutritional conditions in the southern region, in 2019, excess weight reached the highest prevalence in relation to the other outcomes.

Excess weight is the nutritional condition that prevailed for longer periods in relation to other outcomes in the historical series. In the North and Northeast regions, excess weight prevailed from 2012. In the Midwest, it was prevalent from 2011, and it prevailed in the Southeast from 2010. In the South region, excess weight was prevalent from the first year of the analysis (2008) (i.e., over the entire time series).

## Elderly life phase

There was a time trend of decreasing underweight in all regions of Brazil. In 2009, the highest percentage was in the Northeast (21.6%). In 2019, the lowest percentages were in the South (8.9%) and Midwest (11.6%).

Eutrophy showed a decreasing temporal trend in all regions, with its highest prevalence in 2008 in the North (43%) and lowest prevalence in 2019 in the South (32.5%), Midwest (35.5%) and Southeast (36.5%), as shown in Table 6.

**Table 6:** Prevalence and annual time trend of nutritional status of elderly people by Brazilian region from 2008 to 2019. There is a 95% confidence level

Regions	Nutritional status	Ano												*Annual average variation	**P value	Trend
		**2008**	**2009**	**2010**	**2011**	**2012**	**2013**	**2014**	**2015**	**2016**	**2017**	**2018**	**2019**			
		**%**	**%**	**%**	**%**	**%**	**%**	**%**	**%**	**%**	**%**	**%**	**%**			
North	Low weight	20.1	20.3	18.5	18.8	19.0	17.2	17.5	13.8	14.0	13.4	12.7	12.5	-0.795	< 0,001	Decrease
	Eutrophy	43.0	42.9	42.2	41.6	41.2	40.5	39.9	38.5	38.7	38.5	38.1	38.0	-0.510	< 0,001	Decrease
	Excess of weight	36.9	36.8	39.3	39.6	39.9	42.2	42.6	47.7	47.3	48.0	49.2	49.5	1.305	< 0,001	Increase
Northeast	Low weight	20.4	21.6	19.5	18.6	17.7	19.8	17.5	15.6	15.4	14.8	13.9	13.9	-0.694	< 0,001	Decrease
	Eutrophy	40.7	41.7	40.4	41.1	41.0	41.5	40.0	40.3	40.5	40.2	39.6	39.8	-0.132	< 0,001	Decrease
	Excess of weight	38.9	36.6	40.1	40.3	41.3	38.7	42.5	44.1	44.1	45.0	46.6	46.3	0.826	< 0,001	Increase
Midwest	Low weight	19.2	17.4	17.4	15.6	14.7	15.5	15.2	13.0	12.8	12.8	12.2	11.6	-0.634	< 0,001	Decrease
	Eutrophy	39.9	39.7	39.5	39.3	37.9	38.0	36.5	34.3	35.1	35.9	35.6	35.5	-0.500	< 0,001	Decrease
	Excess of weight	40.9	43.0	43.1	45.1	47.4	46.5	48.3	52.8	52.1	51.3	52.2	52.9	1.135	< 0,001	Increase
Southeast	Low weight	19.6	20.0	18.2	17.4	16.4	17.4	17.0	14.4	14.0	14.1	13.5	13.0	-0.639	< 0,001	Decrease
	Eutrophy	38.3	39.5	38.8	38.5	38.2	38.6	38.6	37.3	37.1	37.2	36.8	36.5	-0.227	< 0,001	Decrease
	Excess of weight	42.1	40.5	43.0	44.1	45.4	44.0	44.4	48.3	49.0	48.7	49.7	50.5	0.866	< 0,001	Increase
South	Low weight	13.1	13.7	12.6	10.9	10.6	10.6	11.3	9.6	10.5	9.7	8.9	8.9	-0.397	< 0,001	Decrease
	Eutrophy	36.4	36.5	36.8	35.0	34.3	33.2	32.5	32.8	33.8	33.2	32.6	32.5	-0.402	< 0,001	Decrease
	Excess of weight	50.5	49.8	50.6	54.2	55.1	56.2	56.2	57.6	55.7	57.1	58.5	58.6	0.799	< 0,001	Increase

*Note*: * Annual average variation is calculated by the linear regression method; **P value indicates the results of the two-sided t test

Table 6 Prevalence and annual time trend of nutritional status of elderly people by Brazilian region from 2008 to 2019. There is a 95% confidence level.

In the excess-weight nutritional condition, there was a temporal trend of increase in all regions, with higher percentages in 2019 in the South (58.6%), Midwest (52.9%) and Southeast (50.5%) regions.

In the South, Southeast and Midwest regions, excess weight was higher than the other outcomes throughout the time series (2008 to 2019), with the highest annual prevalence in the South region.

## Pregnancy life phase

In the pregnancy life stage, according to Table 7, there was a decrease in the time trend of the nutritional condition low weight in all regions. There was a higher prevalence of low weight in the South (12.6%) and Southeast (15.4%) regions in 2019 and a higher prevalence in the North (27.6%) and Northeast (26.3%) regions in 2008.

**Table 7:** Prevalence and annual time trend of nutritional status of pregnant by Brazilian region from 2008 to 2019. There is a 95% confidence level

Regions	Nutritional status	Ano												*Annual average variation	**P value	Trend
		**2008**	**2009**	**2010**	**2011**	**2012**	**2013**	**2014**	**2015**	**2016**	**2017**	**2018**	**2019**			
		**%**	**%**	**%**	**%**	**%**	**%**	**%**	**%**	**%**	**%**	**%**	**%**			
North	Low weight	27.6	27.7	27.4	25.1	24.2	24.3	23.2	21.0	21.0	19.6	18.5	18.3	-0.949	< 0,001	Decrease
	Eutrophy	46.2	47.0	46.6	45.9	45.1	44.0	43.6	42.7	42.4	42.2	40.8	39.2	-0.657	< 0,001	Decrease
	Excess of weight	26.3	25.3	26.0	29.0	30.7	31.8	33.1	36.3	36.7	38.2	40.6	42.5	1.605	< 0,001	Increase
Northeast	Low weight	26.3	25.4	23.6	22.2	21.4	21.7	20.7	18.8	18.9	18.4	17.1	16.8	-0.841	< 0,001	Decrease
	Eutrophy	44.7	44.6	44.5	43.0	42.2	41.4	40.7	40.3	39.7	38.9	37.5	36.3	-0.762	< 0,001	Decrease
	Excess of weight	29.0	30.1	32.0	34.8	36.4	36.9	38.5	40.9	41.4	42.7	45.4	46.9	1.604	< 0,001	Increase
Midwest	Low weight	25.3	22.9	22.4	21.1	19.6	19.7	20.3	18.1	17.4	18.3	17.3	17.2	-0.670	< 0,001	Decrease
	Eutrophy	41.4	42.3	42.1	40.7	40.7	39.4	38.0	37.0	37.5	37.2	36.2	35.2	-0.644	< 0,001	Decrease
	Excess of weight	33.3	34.8	35.5	38.2	39.8	40.9	41.8	44.9	45.1	44.5	46.5	47.6	1.314	< 0,001	Increase
Southeast	Low weight	23.3	22.6	20.5	19.0	19.0	19.4	18.9	17.8	17.6	16.3	15.4	15.4	-0.673	< 0,001	Decrease
	Eutrophy	41.4	40.3	40.3	39.4	39.2	38.7	37.8	37.2	36.6	35.8	34.6	33.8	-0.654	< 0,001	Decrease
	Excess of weight	35.3	37.1	39.2	41.6	41.8	42.0	43.3	45.0	45.7	47.9	50.0	50.8	1.327	< 0,001	Increase
South	Low weight	18.1	17.9	17.3	16.6	16.0	15.9	16.0	15.0	14.4	13.7	12.6	12.6	-0.515	< 0,001	Decrease
	Eutrophy	43.4	42.2	42.0	40.4	39.5	38.7	37.6	37.0	37.0	36.0	34.6	33.9	-0.846	< 0,001	Decrease
	Excess of weight	38.5	39.9	40.7	43.0	44.5	45.3	46.4	48.0	48.6	50.3	52.8	53.5	1.361	< 0,001	Increase

*Note*: * Annual average variation is calculated by the linear regression method; **P value indicates the results of the two-sided t test

Table 7 Prevalence and annual time trend of nutritional status of pregnant women by Brazilian region from 2008 to 2019. There is a 95% confidence level.

The research showed that in the pregnancy phase of life, eutrophy showed a decrease in the time trend in all regions. The highest prevalence of eutrophy occurred in the North (47,0%) in 2009, and the lowest prevalence was in the Southeast (33.8%) and South (33.9%).

In the excess-weight nutritional condition, there was a temporal trend of increase in all regions, with higher percentages in 2019 in the South (53.5%) and Southeast (50.8%).

In the South and Southeast regions, the prevalence of excess weight has predominated over eutrophy in the historical series since 2011.

Pregnant women are a group with greater biological vulnerability, and monitoring their nutritional status is of great importance.

## Discussion

When analysing the prevalence of excess weight in children, one finds a scenario of nutritional transition in which malnutrition (thinness and marked thinness), which until then was responsible for high infant mortality rates worldwide and in Brazil, gave way to infant obesity, including in the age group in which breastfeeding should reflect in an appropriate nutritional state that is eutrophic (0 to < 2 years old). At this stage, obesity implies decreased generational life expectancy [17]. Additionally, in the child life stage from 5 to < 10 years old, there was the most critical prevalence of excess weight.

However, in the stages of children's lives, malnutrition persisted, even if the prevalence was low, especially in the Northeast.

Research [35] has indicated that child malnutrition could have ceased to be a public health problem over the last decade in Brazil through the maintenance of economic and social policies that allow access to essential services and increase the incomes of the poorest people. However, this situation did not materialize; thinness and accentuated thinness, although reduced, have persisted as a public health problem in more vulnerable regions [36]. Additionally, the “dynamics of the double burden of malnutrition” [4] show that stunting, weight loss and thinness have decreased, whereas excess weight has increased. A nutritional transition was also identified and attributed to changes in the food system that are responsible for altering children's diets, such as the availability of ultra-processed, low-nutrition and inexpensive foods and beverages in low- and middle-income countries [4]. In Brazil, through assistance in Basic Health Units (BHUs), the Family Health Strategy (FHS) had a positive impact on the drop in infant mortality levels [37, 38].

In the pregnancy life phase, the time trend of increasing excess weight in all regions demonstrates a growing health problem among women of reproductive age. According to one study [39], excess weight in early pregnancy was associated with increased BMI and greater odds of obesity in young adults as well as established risk factors for adverse pregnancy, delivery and birth outcomes [40].

In the adolescent life phase, there was a decreasing trend in the eutrophic nutritional condition due to increases in the excess weight percentiles.

According to studies [2, 41, 42], the prevalence of obesity in children and adolescents has been identified in several countries, reinforcing the notion that public policies should encourage the consumption of healthy food and access to this food by the poorest people. In Brazil, the National School Food Program (NSFP) [43] is considered to be an important step towards promoting food security, especially in the child and adolescent life phases, as it allows access to nutritious, sustainable food produced by family farmers and provided to public schools.

The time series analysis shows increasing trends of excess weight in the regions of Brazil, with the highest percentages in the adult population and in the southern region. Other research from Brazil has also indicated the growth of obesity among adults [18]. National data differ from American studies [44, 45], which have reported stabilization in obesity trends in both adults and children.

Research in Australia [46] and the United States [47] has associated overweight and obesity with premature mortality in adults of both sexes, representing an economic cost to health. In the Americas, CNCDs account for three out of four deaths, with 34% considered premature mortality. In funding the burden of CNCDs, low- and middle-income countries reflect this impact in socioeconomic inequalities, as 30% of premature mortality from CNCDs occurs in the poorest population [48, 49].

Thus, the prevalence of excess weight was identified in all life stages and macroregions, with higher percentages compared to thinness and accentuated thinness, indicating that the AI scenario is related to a rapid nutritional transition process that was already evidenced in studies from Latin America [8, 50]. In the adult and elderly life phases, excess weight predominates even over eutrophy, which remains dominant in the life phases of children and adolescents. The findings of the longitudinal series provide data relevant to the objectives of the FNSS to plan actions for public policies of the FNS in the prevention of overweight and obesity.

The coexistence of hunger and malnutrition, micronutrient deficiencies and the prevalence of excess weight (overweight and obesity) occur, especially due to the lack of access to a healthy diet that provides the nutrients needed to promote human health and well-being [4, 51]. Determinant factors have been evidenced in studies [4, 13] addressing the double burden of malnutrition affecting low- and middle-income countries and attributing the phenomenon to rapid changes in food systems and diets globally [52]. They focus on the energy imbalance that causes weight gain, especially due to the intake of ultra-processed foods. Thus, the AI scenario compromises the human rights, real freedoms and opportunities necessary to achieve sustainable development goals [53, 54].

The prevalence of excess weight differs among macroregions, where the aspects of human and territorial development, access and the rights of people to adequate and healthy food are disparate. The prevalence of excess weight is higher in regions with higher Human Development Indices (HDIs) [53] (South, Southeast and Center-West) and lower in regions with lower HDIs (North and Northeast) [55]. It is relevant to consider that demographic distribution, environmental factors, climate, geography, lifestyle, income, education level and food consumption habits can vary according to different countries and regions of the same country; these factors are mentioned in other studies [8, 56].

The deprivation of resources to promote changes in lifestyle [53] and the poorer access and quality of health services, which promote physical and social well-being with actions to prevent risk factors and the difficulty in obtaining a nutritious and healthy diet [57], reinforce a growing scenario of excess weight prevalence in the country [49, 58]. A study by the Institute of Applied Economic Research (IPEA) [59] concluded that obesity reflects the existence of structural inequalities in Brazilian society.

Biological factors and social determinants are involved in the different phases of life. Vulnerability, fragility, premature death and habit building in childhood, adolescents, adults and elderly people, established risk factors for adverse pregnancy, should be considered when discussing FI in relation to excess weight.

The COVID-19 pandemic has worsened the state of food insecurity in Brazil [60] by deepening hunger and economic and social inequalities. Global organizations [61] have reported that the COVID-19 pandemic has intensified all forms of malnutrition, vulnerabilities and inadequacies in global food systems. Thus, far from meeting global nutrition targets, the achievement of Sustainable Development Goal 2 (Zero Hunger) by 2030 has been compromised.

The modernisation of the means of transportation and fast and practical alimentation aimed at meeting the modern profile of work have led to changes in body patterns, which, allied with sedentarism, are constituents of an obesogenic environment. It is believed that these issues of modernity, food systems and social welfare should be part of the agendas for promoting the FNS and obesity control. The existence of public policies that connect the multidimensional aspects of food, good health and nutrition practices with nature are fundamental to the construction of healthy diets [62, 63] with consumers.

The promotion of food and nutritional security requires public policies that exceed the logic of modernised agriculture and support agriculture that is sensitive to nutritional, adequate and healthy foods and appropriate to cultural habits [58, 64–66]. Such assumptions reinforce the syllogism represented by sustainable diets in the control of excess weight. Authors [67, 68] have shown that sustainable diets, called planetary health diets, are essential to human health and environmental sustainability, as they go beyond the nutritional perspective [69] and become a challenge for sustainable food systems [70] in the Anthropocene.

The study innovated using FNSS data to emphasize the prevalence of excess weight. It sought to compare the five macroregions of the country and the stages of life and analysed a 12-year time series. Studies were not found in the literature including such a national scope, analysing such a large database and making these comparisons.

The study contributes to the diagnosis of the nutritional situation and detects nutritional transition, predicting the nutritional conditions of the population monitored in the basic health network and registered in the SISVAN, contributing to the use of the potential of health information in the planning, management and assessment of nutritional status in SUS primary care and strengthening of information for the National Food and Nutrition Security Policy (NFNSP) [71].

As a main conclusion, the study shows that excess weight has increased throughout the entire historical series analysed in Brazil, being highest in the adult population and in the southern region.

## Supplementary Information

**Additional file 1.**

**Additional file 2.**

**Additional file 3.**

## Data Availability

The datasets generated and/or analysed during the current study are available in the [Marcia-Domenica/Nutritional-status-of-Brazilians] repository, https://github.com/Marcia-Domenica/Nutritional-status-of-Brazilians The secondary database of the FNSS (SISVAN) is available at: http://sisaps.saude.gov.br/sisvan/relatoriopublico/index

## Abbreviations

FI: Food Insecurity
FNSS: Food and Nutritional Surveillance System
FNS: Food and Nutritional Surveillance
CNCDs: Chronic noncommunicable diseases
IBGE: Brazilian Institute of Geography and Statistics
FBS: Family Budget Survey
FHS: Family Health Strategy
FNS: Food and Nutrition Security
NFNSP: National Food and Nutrition Security Policy
BHUs: Basic Health Units
UHS: Unified Health System
HIS: Health Information System
HDIs: Human Development Indices
