# Supplementary material for: Characterization of the prevalence of excess weight in Brazil
Source: BMC Public Health. 2022 Jun 6;22:1131. doi: 10.1186/s12889-022-13462-9 (PMC9169593; doi:10.1186/s12889-022-13462-9)
Supplement: Supplementary file 3 — Additional file 3. [file 12889_2022_13462_MOESM3_ESM.pdf]

Figure 1

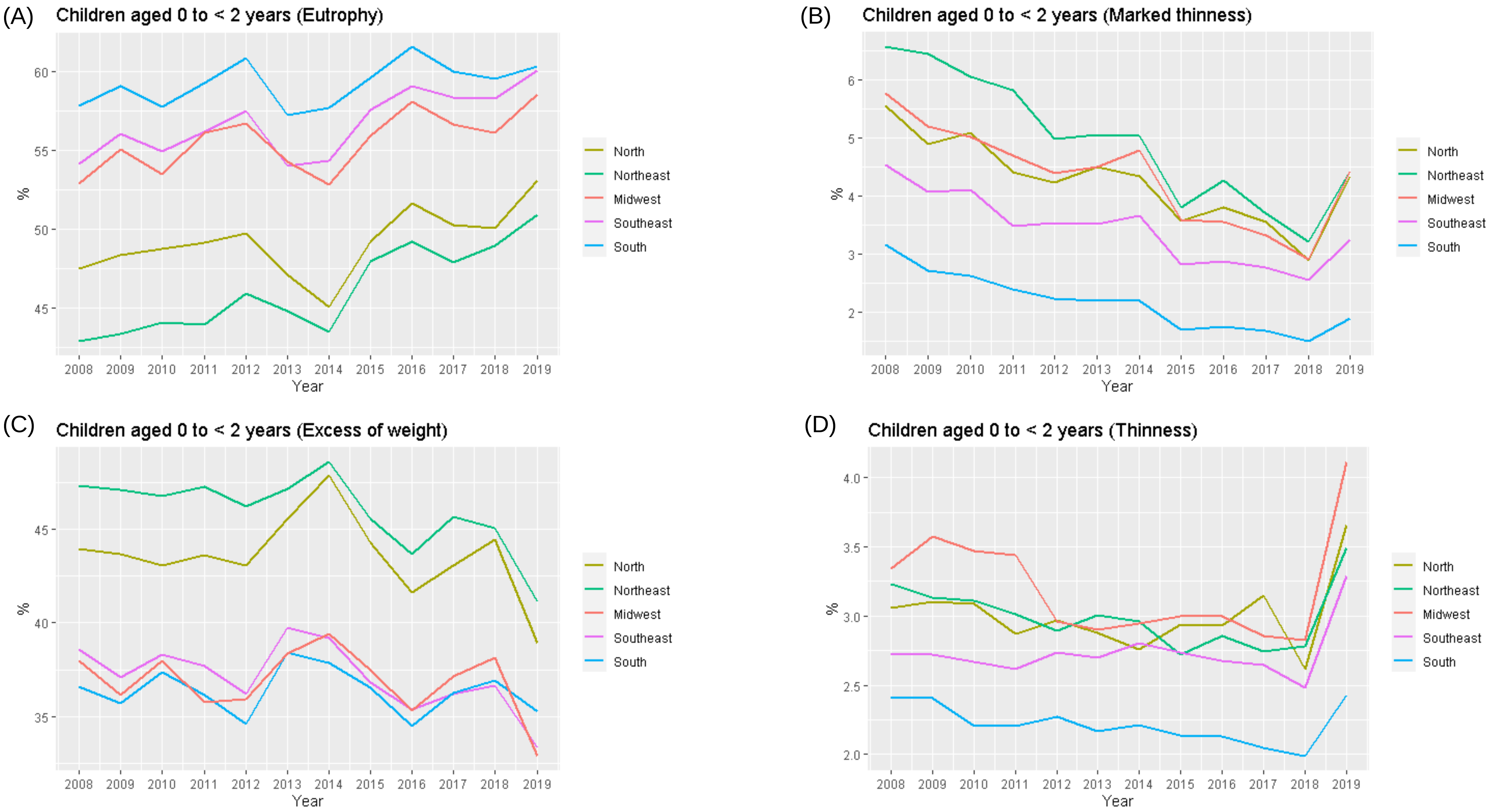

Trends in Child life phase (0 to < 2 years old) by nutritional conditions, Brazil 2008-2019.

Figure 2

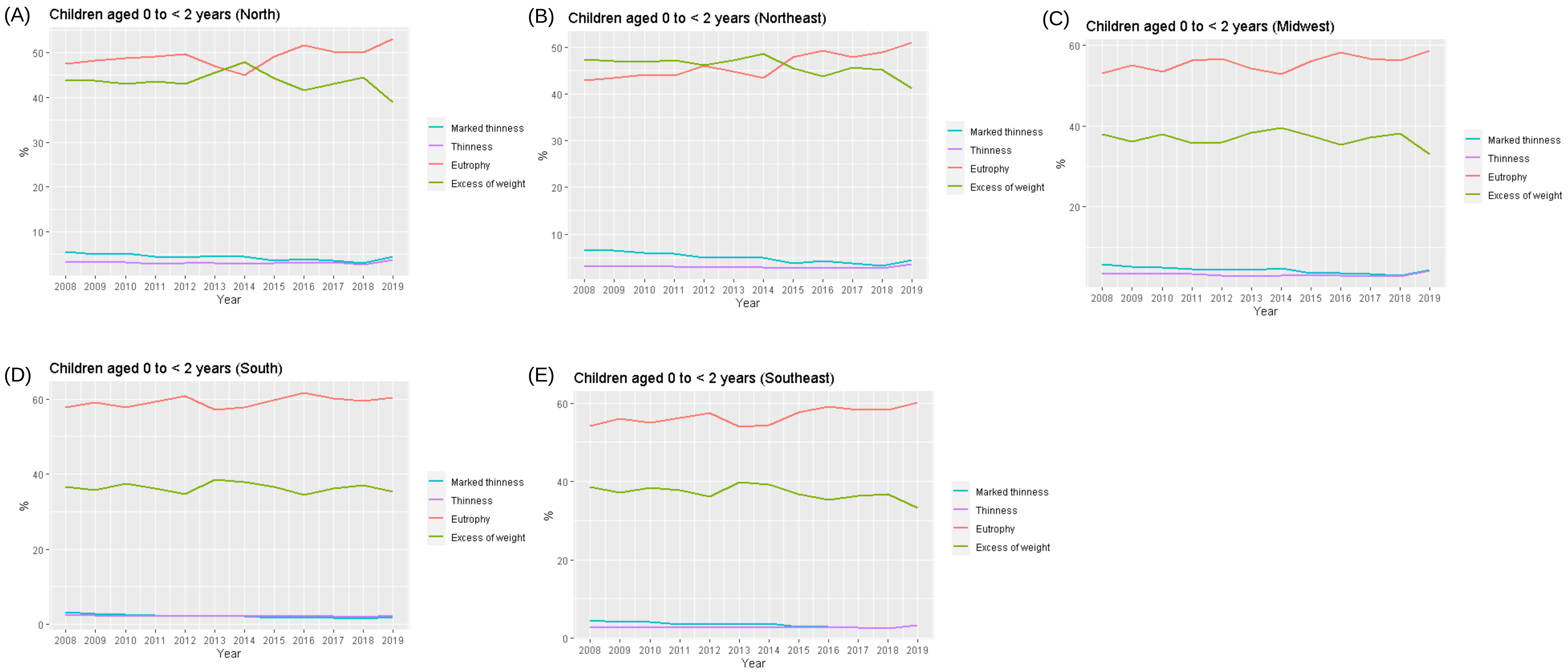

Trends in Child life phase (0 to < 2 years old) by region, Brazil 2008-2019.

Figure 3

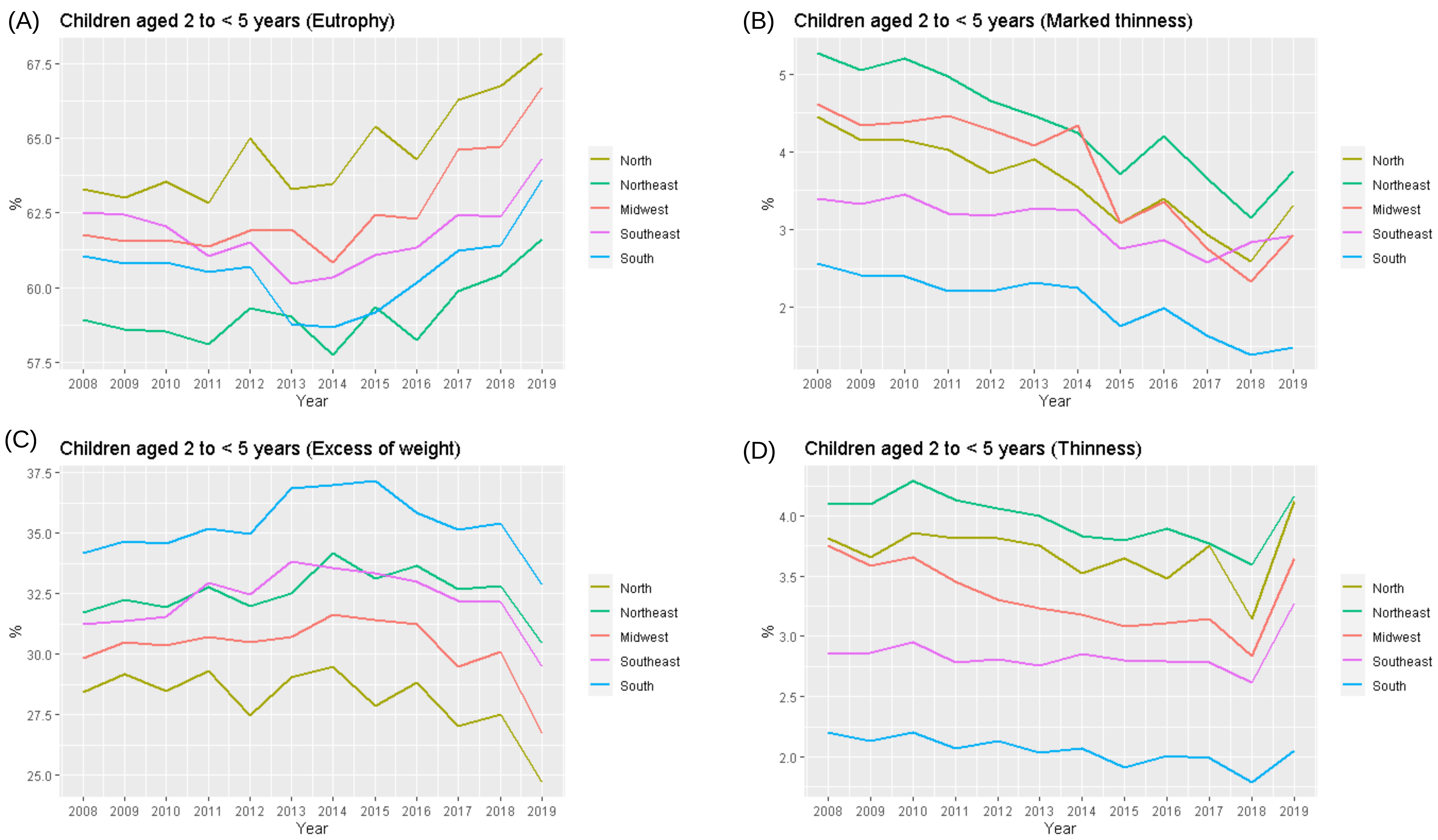

Trends in Child life phase (2 to < 5 years old) by nutritional conditions, Brazil 2008-2019.

Figure 4

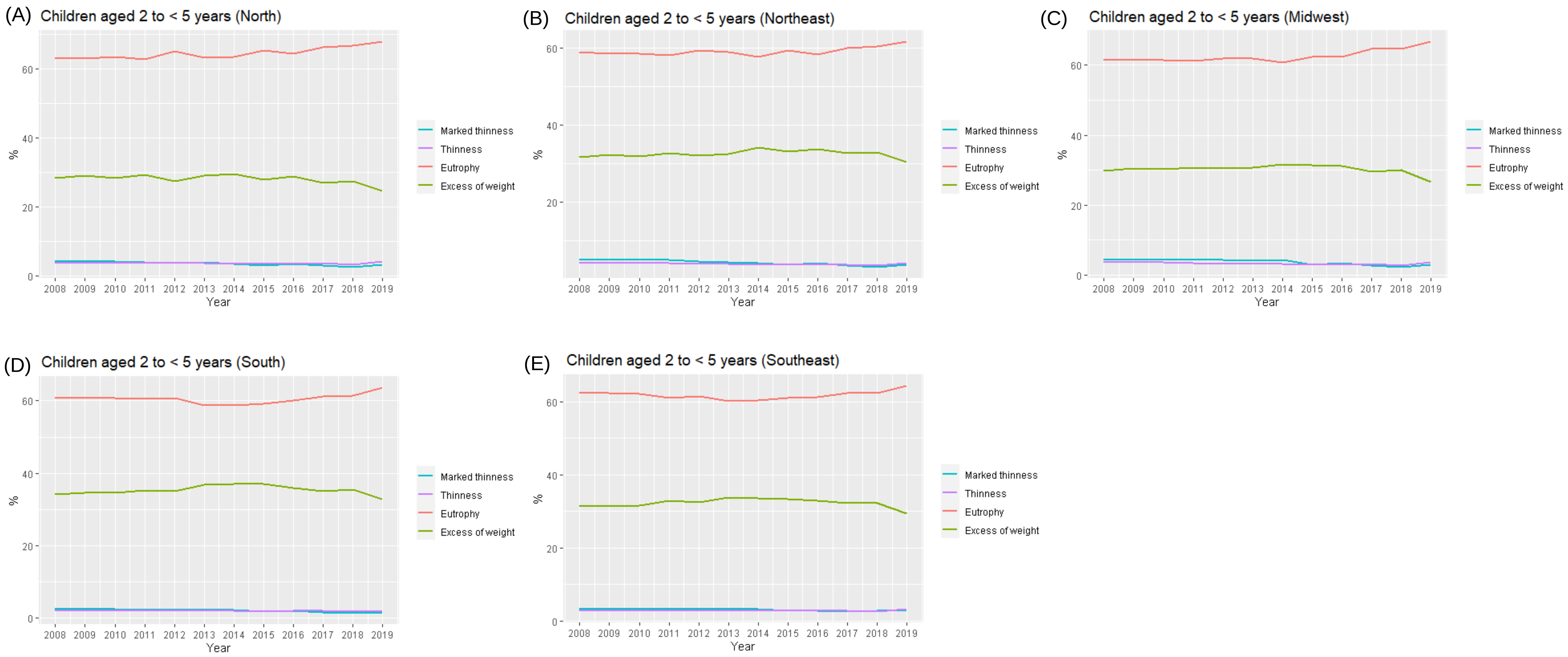

Trends in Child life phase (2 to < 5 years old) by region, Brazil 2008-2019.

Figure 5

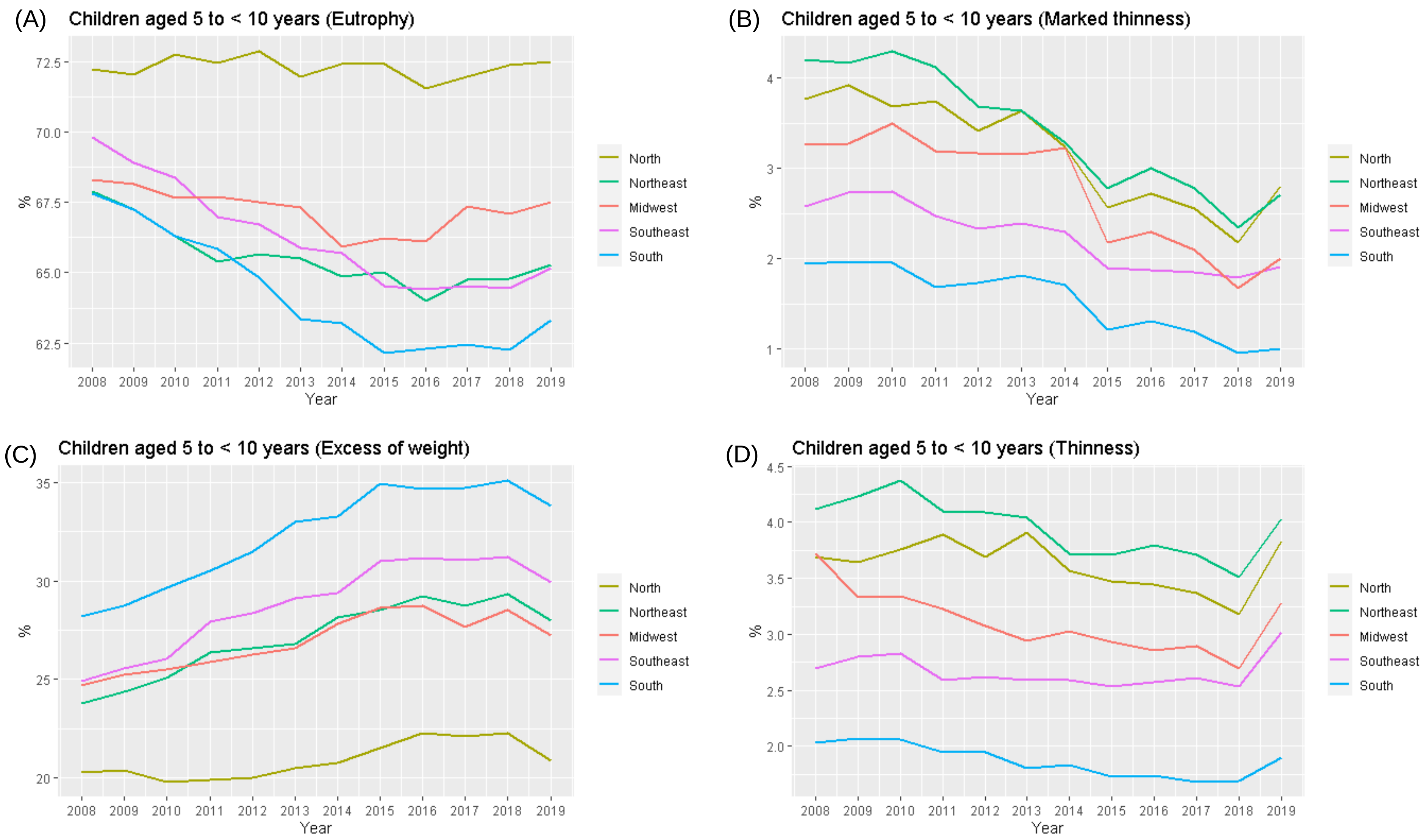

Trends in Child life phase (5 to < 10 years old) by nutritional conditions, Brazil 2008-2019.

Figure 6

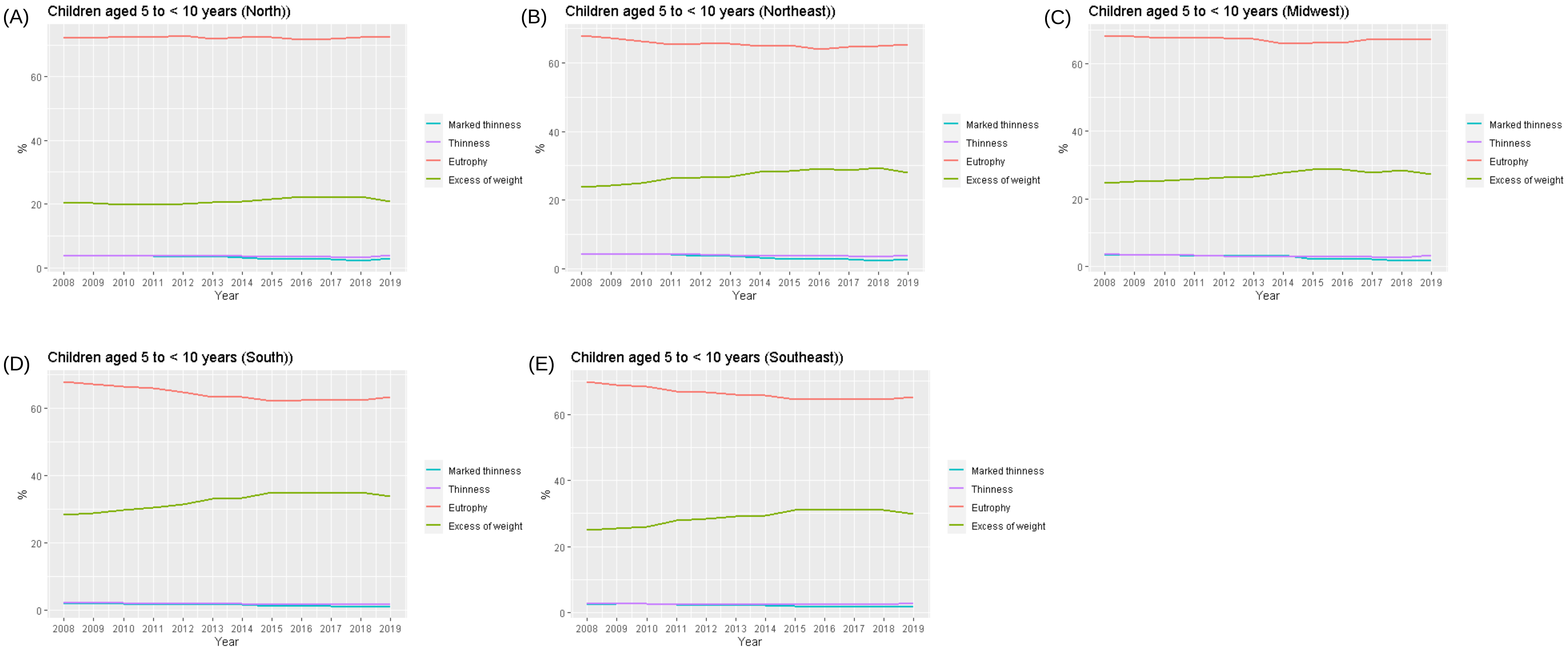

Trends in Child life phase (5 to < 10 years old) by region, Brazil 2008-2019.

Figure 7

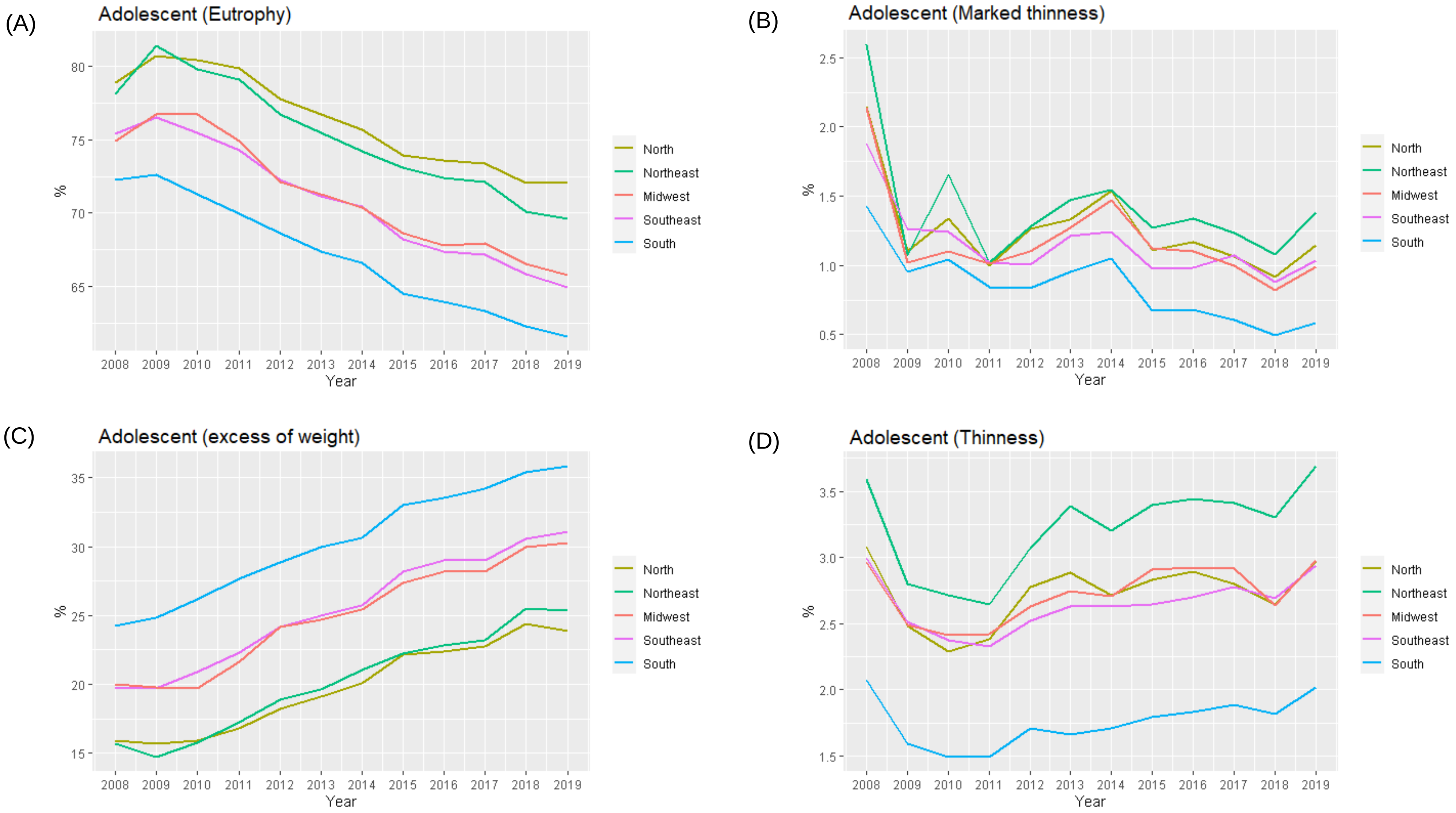

Trends in Adolescent life phase by nutritional conditions, Brazil 2008-2019.

Figure 8

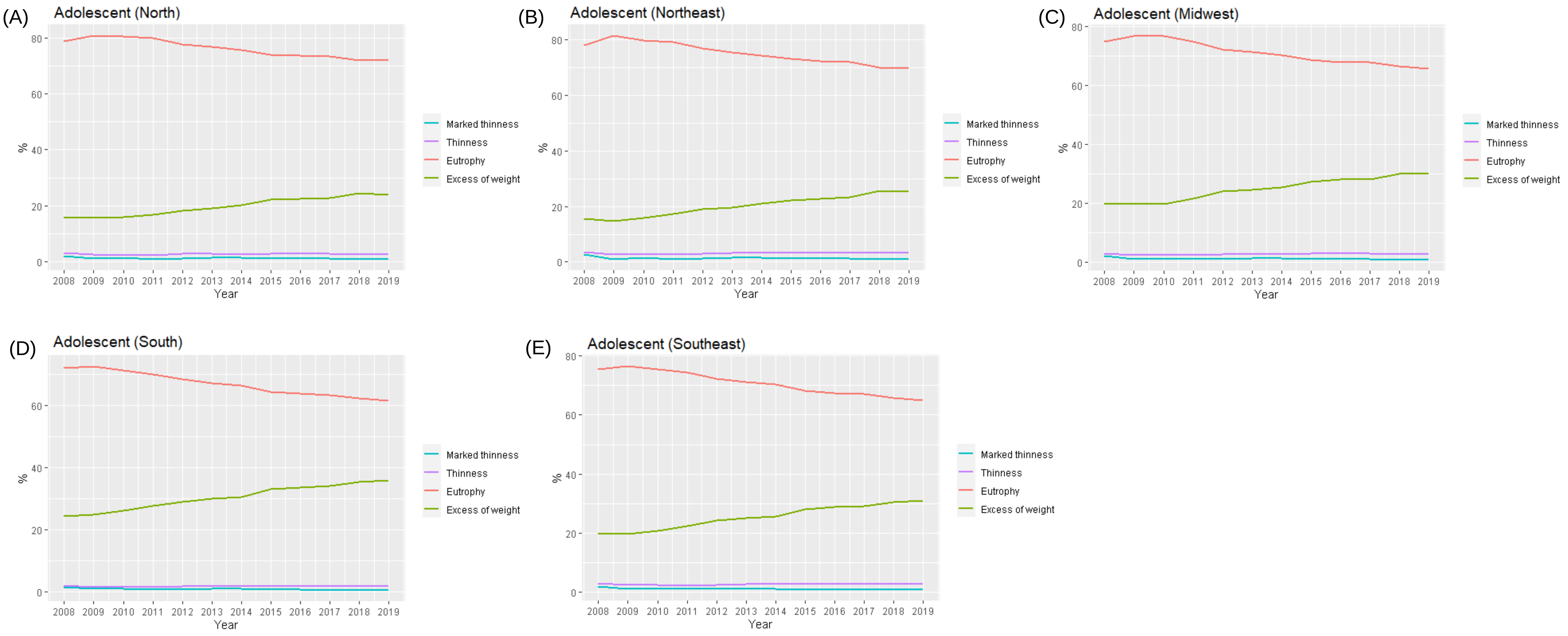

Trends in Adolescent life phase by region, Brazil 2008-2019.

Figure 9

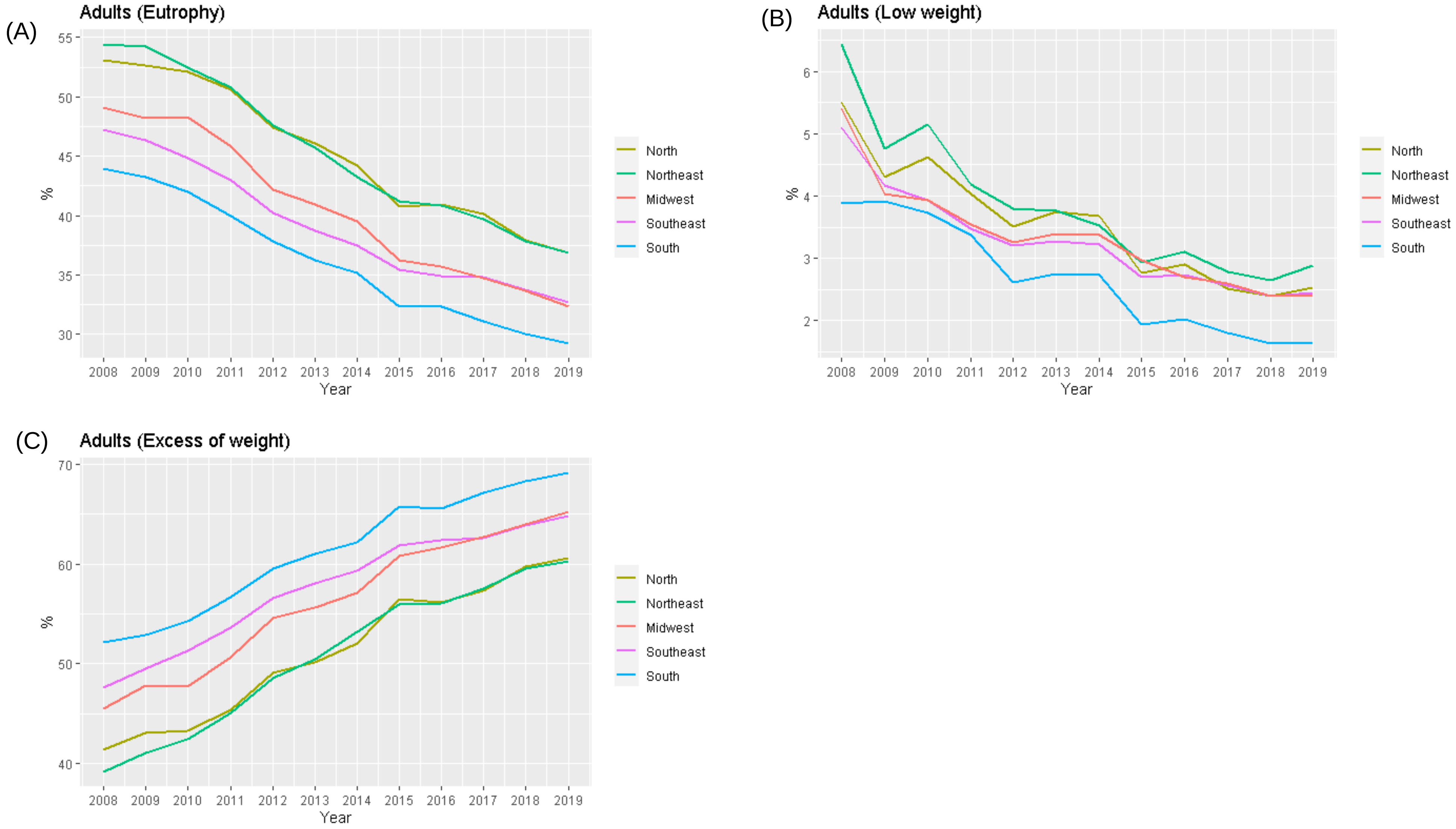

Trends in Adult life phase by nutritional conditions, Brazil 2008-2019.

Figure 10

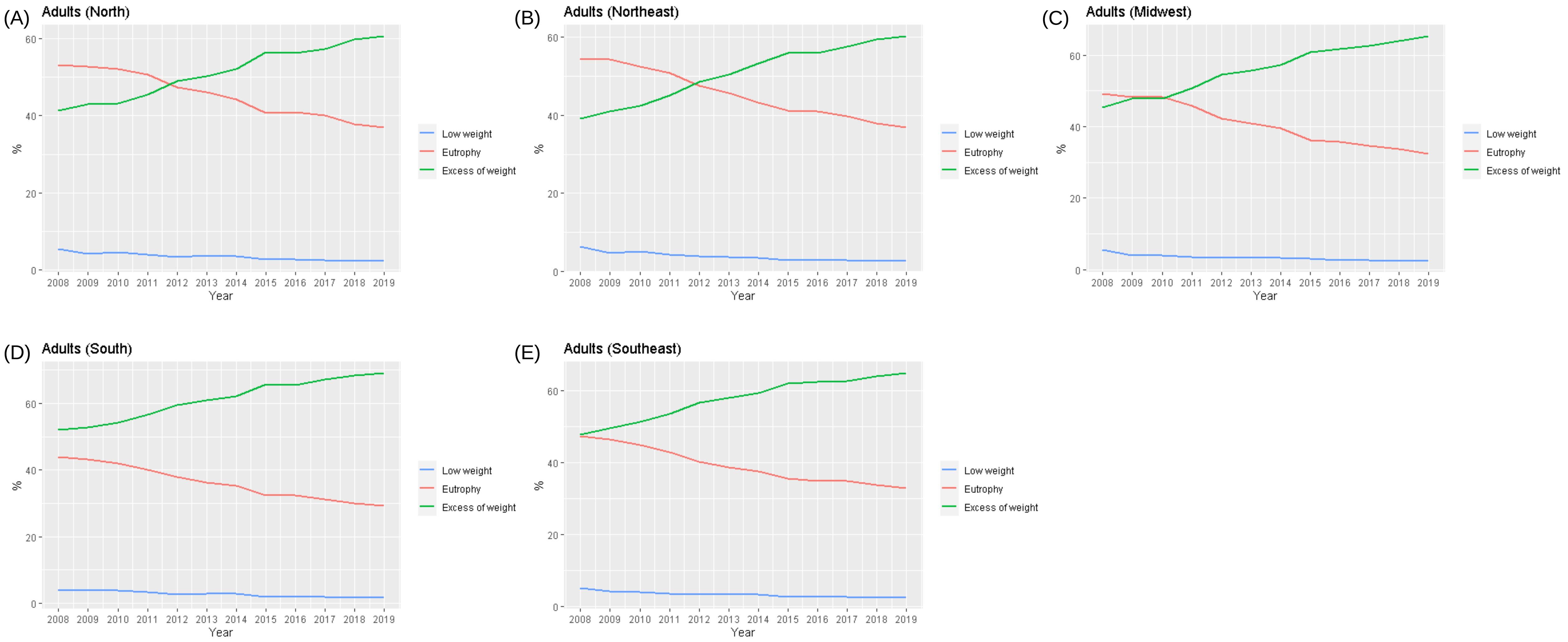

Trends in Adult life phase by region, Brazil 2008-2019.

Figure 11

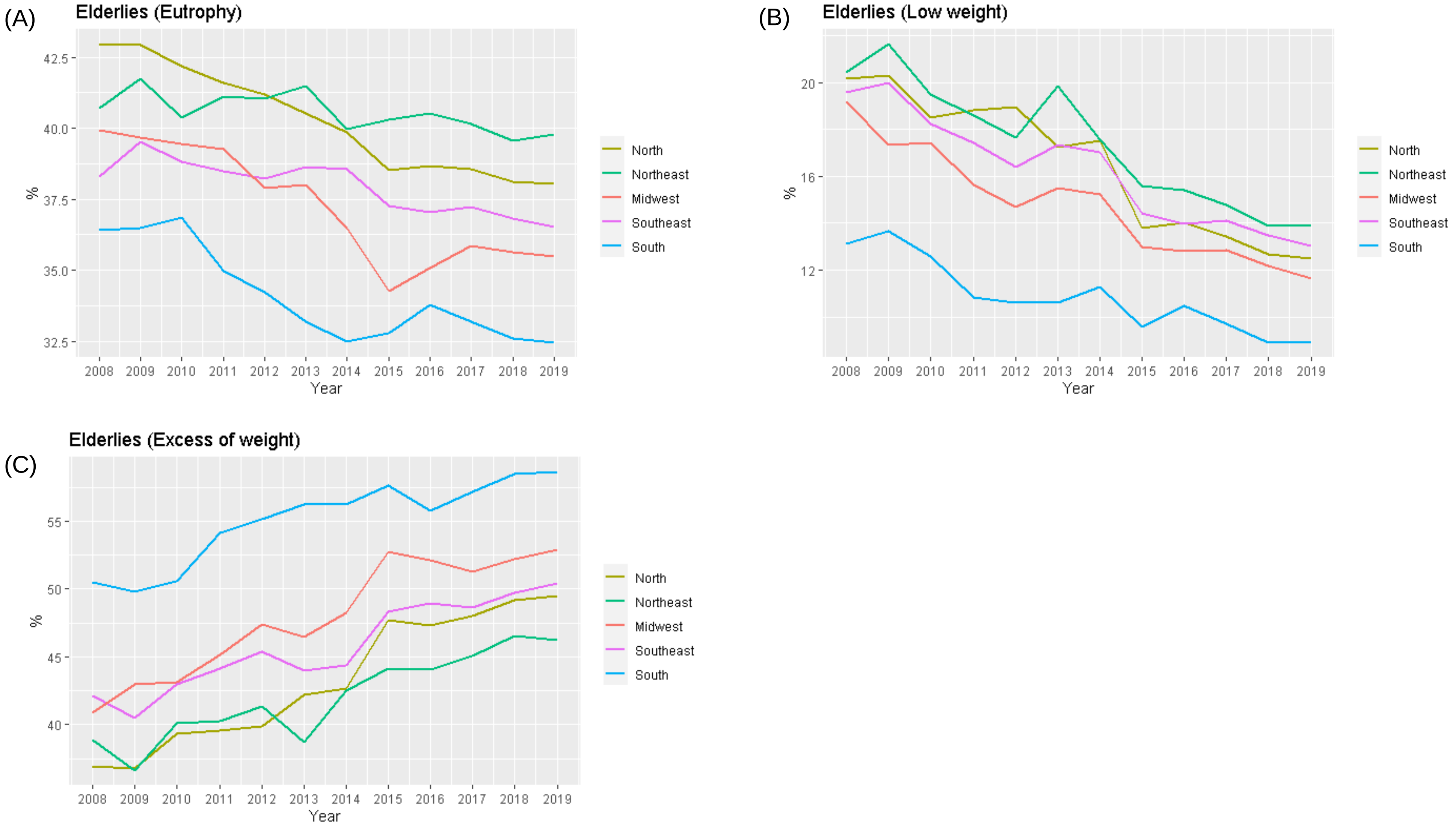

Trends in Elderly life phase by nutritional conditions, Brazil 2008-2019.

Figure 12

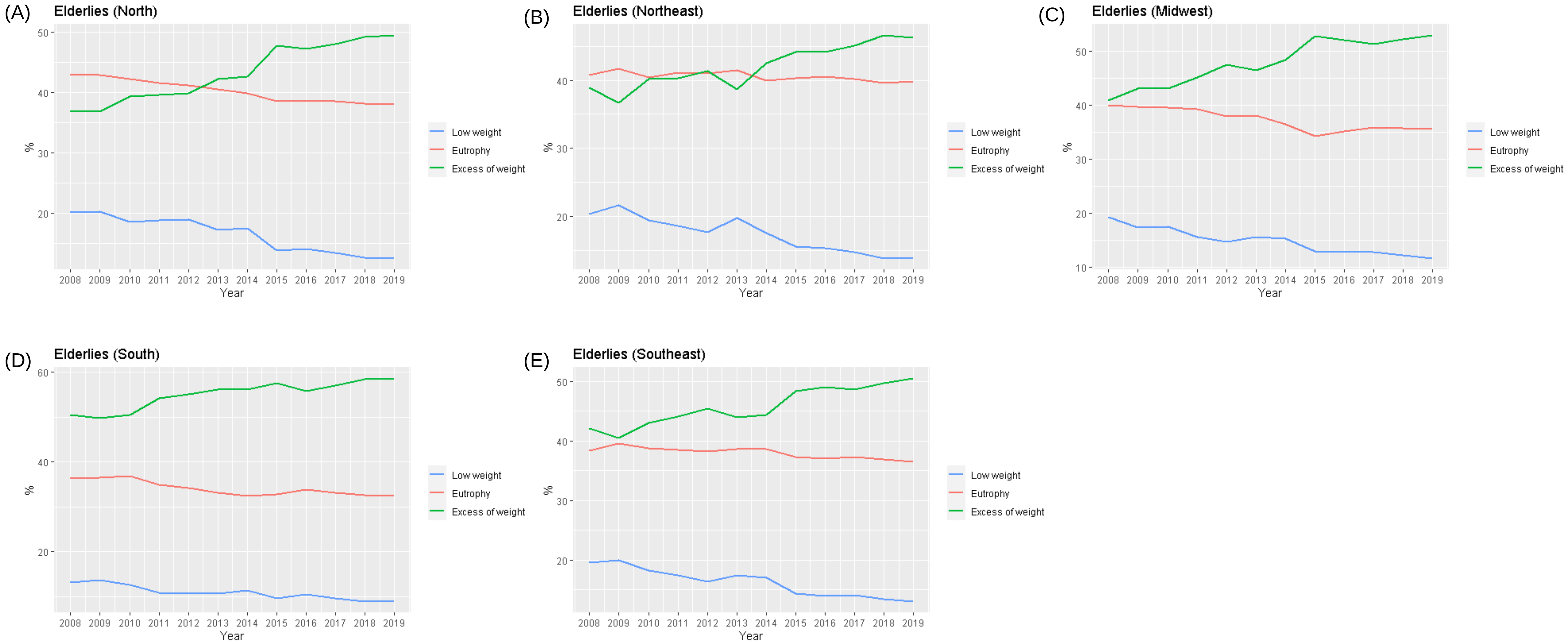

Trends in Elderly life phase by region, Brazil 2008-2019.

Figure 13

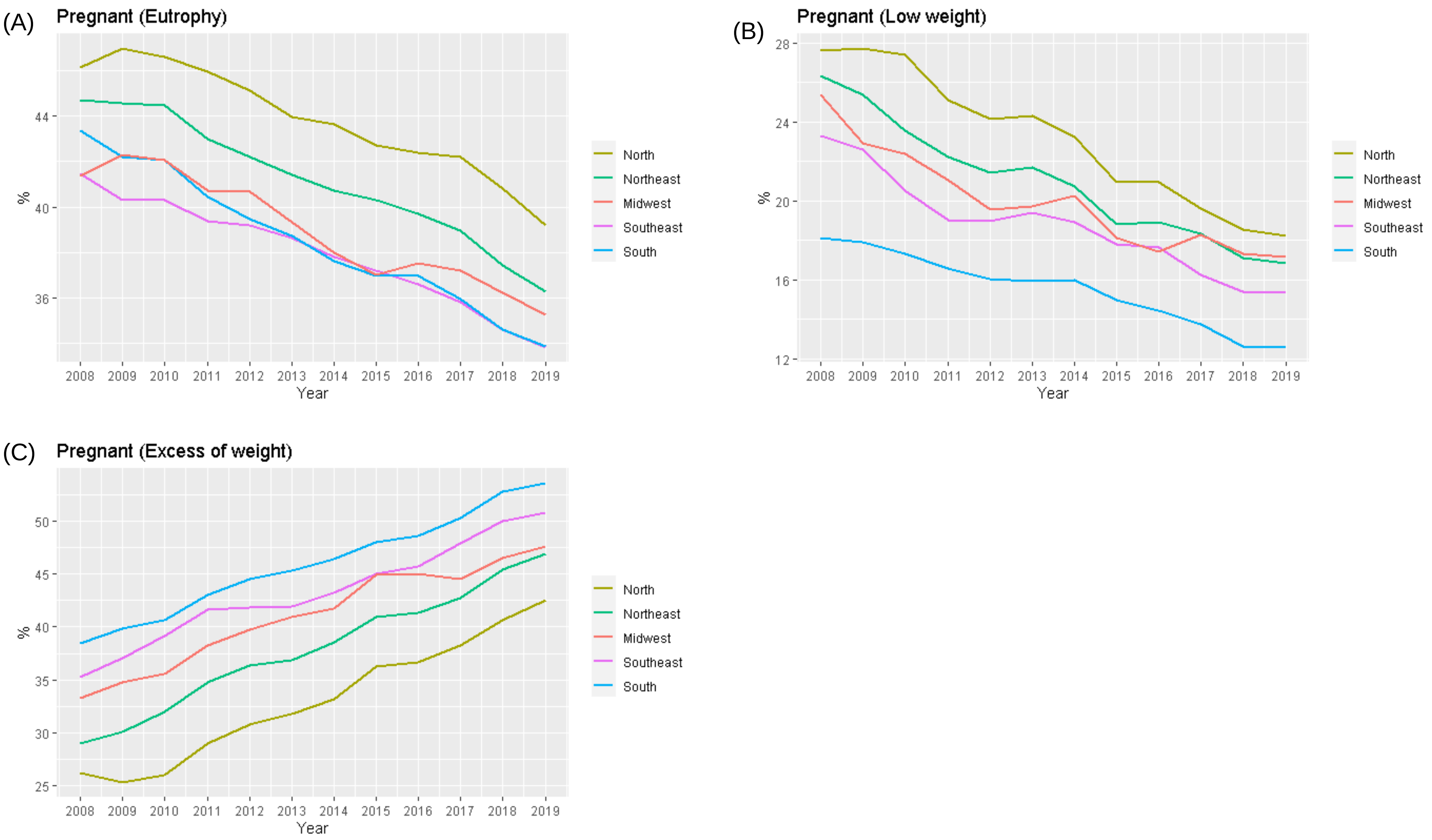

Trends in Pregnancy life phase by nutritional conditions, Brazil 2008-2019.

Figure 14

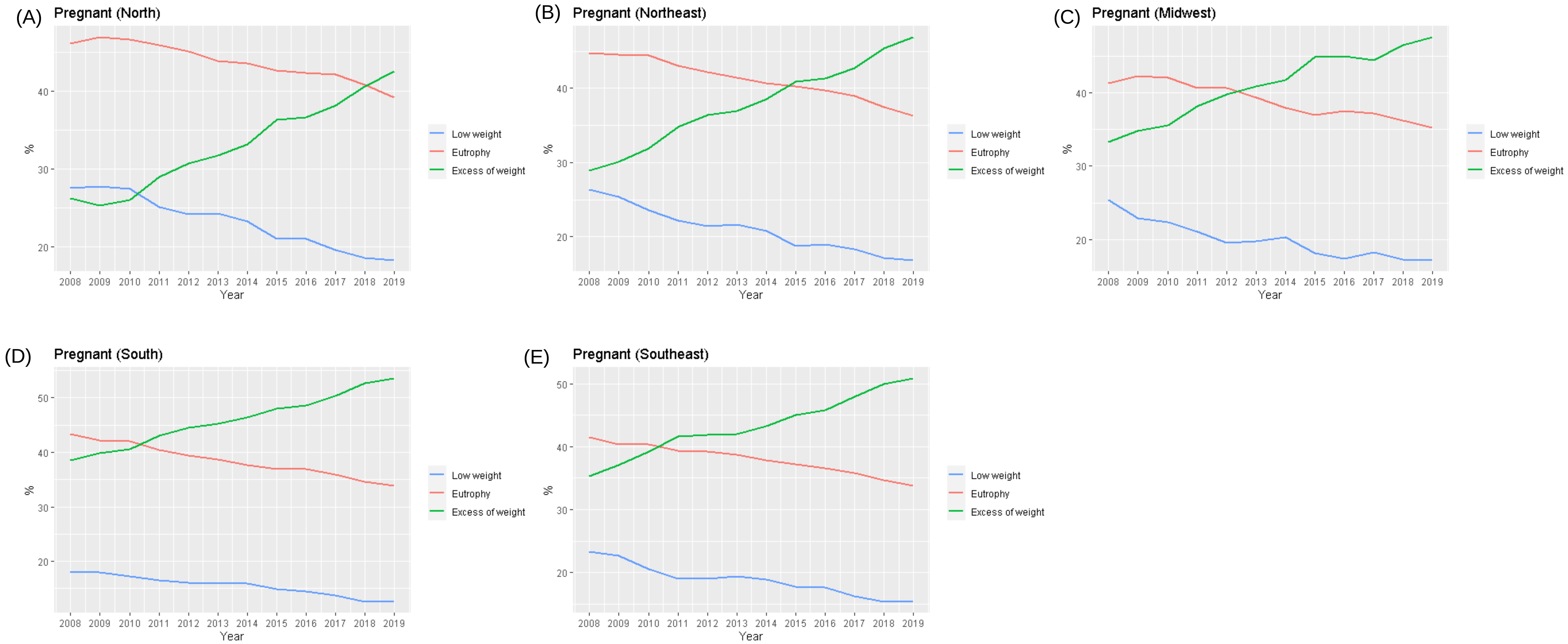

Trends in Pregnancy life phase by region, Brazil 2008-2019.

Figure 15

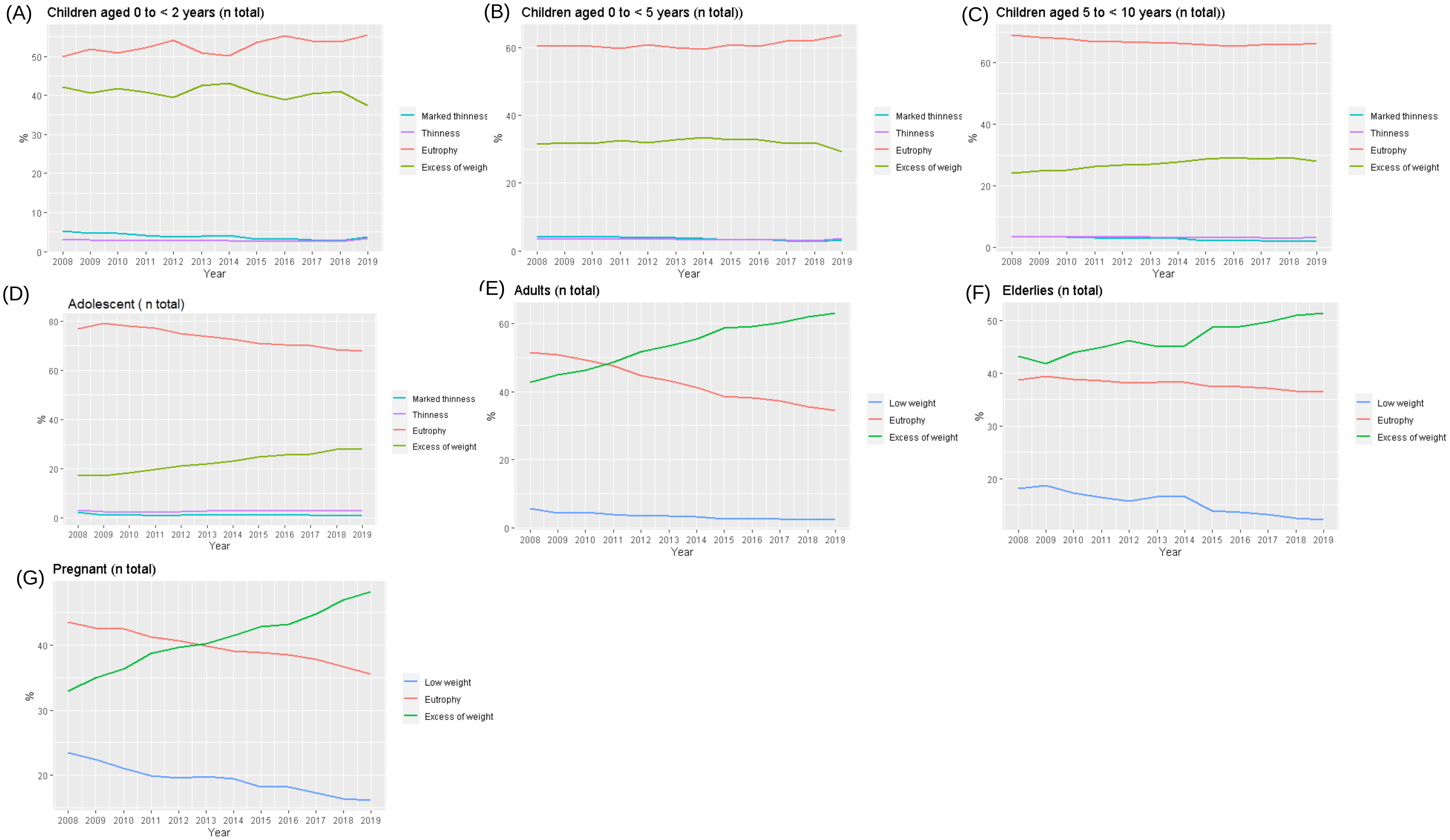

Trends in nutritional status, by life stage of the total number of people monitored by SISVAN in Brazil (n total of the study), Brazil 2008-2019.
